# Myeloid cell-derived apCAFs promote HNSCC progression by regulating proportion of CD4^+^ and CD8^+^ T cells

**DOI:** 10.1186/s13046-025-03290-1

**Published:** 2025-01-31

**Authors:** Feilong Ren, Lin Meng, Shize Zheng, Jiasen Cui, Shaoyi Song, Xing Li, Dandan Wang, Xing Li, Qilin Liu, Wenhuan Bu, Hongchen Sun

**Affiliations:** 1 Hospital of Stomatology, Jilin University, Changchun, 130021 China; 2 Jilin Provincial Key Laboratory Oral Biomedical Engineering, Jilin University, Changchun, 130021 China; 3 Jilin Provincial Key Laboratory of Tooth Development and Bone Remodeling, Jilin University, Changchun, 130021 China; 4 Department of Oral and Maxillofacial Surgery, Hospital of Stomatology, Jilin University, Changchun, 130021 China; 5 School and Hospital of Stomatology, China Medical University, Shenyang, 110002 China

## Abstract

It is well-known that cancer-associated fibroblasts (CAFs) are involved in the desmoplastic responses in Head and Neck Squamous Cell Carcinoma (HNSCC). CAFs are pivotal in the tumor microenvironment (TME) molding, and exert a profound influence on tumor development. The origin and roles of CAFs, however, are still unclear in the HNSCC, especially antigen-presenting cancer-associated fibroblasts (apCAFs). Our current study tried to explore the origin, mechanism, and function of the apCAFs in the HNSCC. Data from single-cell transcriptomics elucidated the presence of apCAFs in the HNSCC. Leveraging cell trajectory and Cellchat analysis along with robust lineage-tracing assays revealed that apCAFs were primarily derived from myeloid cells. This transdifferentiation was propelled by the macrophage migration inhibitory factor (MIF), which was secreted by tumor cells and activated the JAK/STAT3 signaling pathway. Analysis of the TCGA database has revealed that markers of apCAFs were inversely correlated with survival rates in patients with HNSCC. In vivo experiments have demonstrated that apCAFs could facilitate tumor progression. Furthermore, apCAFs could modulate ratio of CD4^+^ T cells/CD8^+^ T cells, such as higher ratio of CD4^+^ T cells/CD8^+^ T cells could promote tumor progression. Most importantly, data from in vivo assays revealed that inhibitors of MIF and p-STAT3 could significantly inhibit the OSCC growth. Therefore, our findings show potential innovative therapeutic approaches for the HNSCC.

**Significance**: ApCAFs derived from myeloid cells promote the progression of HNSCC by increasing the ratio of CD4^+^/CD8^+^ cells, indicating potential novel targets to be used to treat the human HNSCC.

**Graphical Abstract:**

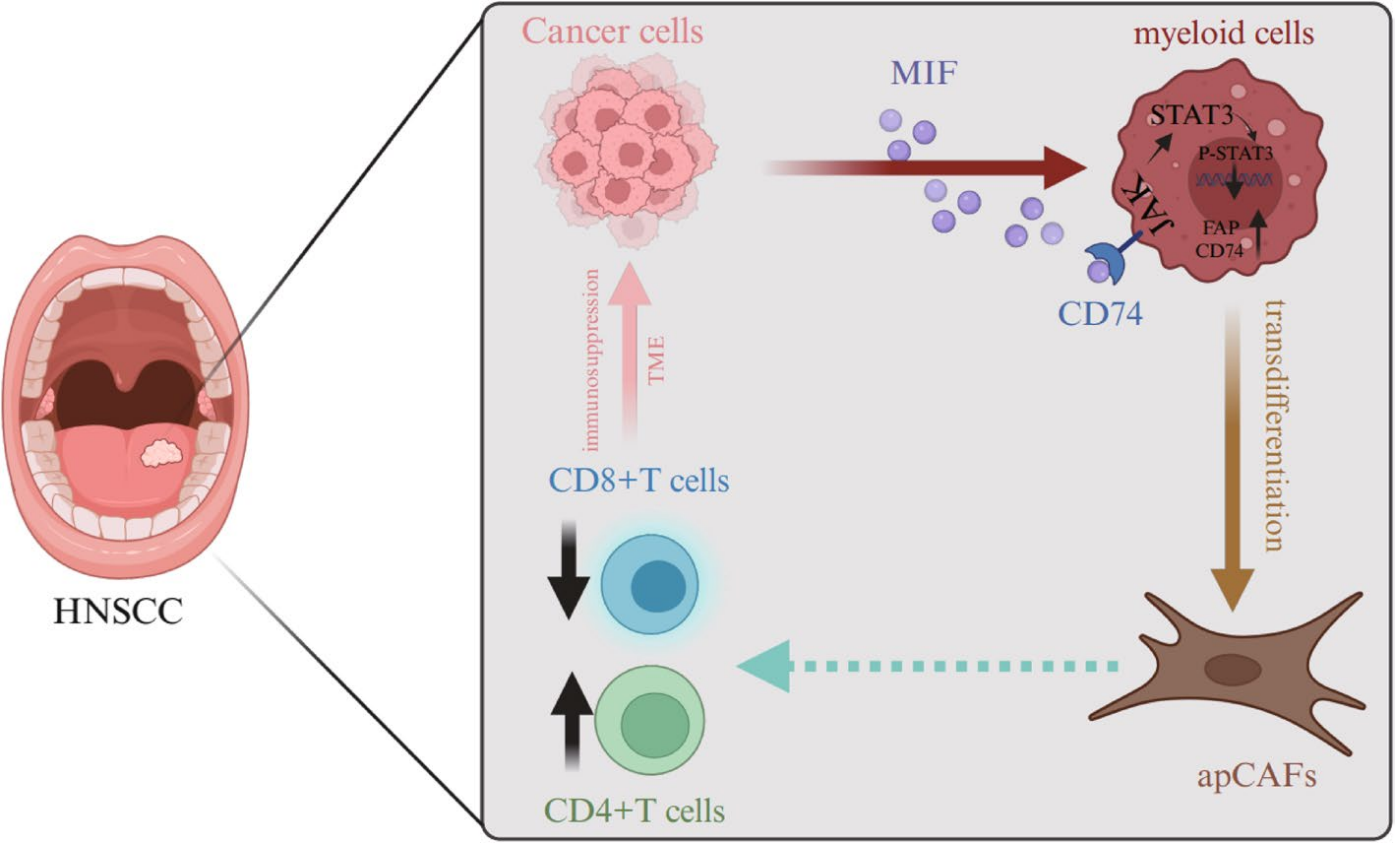

**Supplementary Information:**

The online version contains supplementary material available at 10.1186/s13046-025-03290-1.

## Introduction

HNSCC occurs in the head and neck epithelium, including the oral cavity, pharynx, and larynx [1]. Annually, it has approximately 600,000 new cases of the HNSCC worldwide [2]. The high incidence of HNSCC is closely linked to various factors, such as smoking, alcohol consumption, human papillomavirus (HPV) infection, betel nut chewing, and chronic inflammatory conditions [3]. Despite advancements in its treatment approaches, like surgery, radiotherapy, chemotherapy, and immunotherapy, the 5-year survival rate has shown minimal improvement over the last three decades [4]. The prognosis for HNSCC patients remains bleak due to rapid progression to lymph node metastasis, local recurrence and a high rate of resistance to treatment [5]. New therapeutic treatments for the HNSCC are needed.

TME acts as the vital necessary foundation for the survival and progression of tumors, which consists of various cell types, such as tumor cells, immune cells, stromal cells, and many acellular components, such as extracellular matrix (ECM) and cytokines [6]. All these factors not only coexist, but also dynamically interact resulting in profoundly influencing the tumor biological behaviors and functions, like its initiation, development, infiltration, metastasis, immune evasion, and responses to treatments [7]. To study cancer, the understanding of the entire TME becomes the most important step, especially for the HNSCC that interstitial fibrosis within the TME is one of the most pronounced malignancy responses [8]. Therefore, the best understandings of the TME are essential to develop targeted therapeutic strategies, navigate the complex TME landscape, and ultimately enhance HNSCC treatment outcomes.

CAFs, a predominant cell type in the TME, play vital roles in the development of HNSCC [9]. They contribute to the formation of tumor-permissive ECM, angiogenesis, immunosuppression and metabolic reprogramming of the cell components of TME, accelerating metastasis and resistance to radiotherapy and chemotherapy [10]. The diverse and context-dependent effects of CAFs on carcinogenesis reflect their heterogeneity and plasticity. Attempts to deplete α-SMA^+^ CAFs or inhibit stromal proliferation have been shown to reduce survival times in the mouse model of pancreatic ductal adenocarcinoma (PDAC) [11]. In 2021, two primary clusters of CAFs, inflammatory CAFs (iCAF) and myofibroblasts (myCAF) were identified according to distinct cellular specificity markers and functions [12]. Recent studies indicated that CD105 can be used to distinguish the lineage of CAFs between two pancreatic fibroblast lineages, which CD105^−^ fibroblasts restrict tumor growth via a conventional dendritic cell-dependent mechanism [13]. The MHC-I^hi^Gal9^+^ CAFs are inversely related to the presence of a TCF1^+^GZMK^+^ subset of CD8^+^ T cells, and Gal9 on CAFs induce CD8^+^ T-cell dysfunction and decrease tumor-infiltrating TCF1^+^CD8^+^ T cells in the TME of HNSCC [14]. The CAFs may be differentiated from local fibroblasts, pericytes, mesenchymal stem cells, bone marrow hematopoietic stem cells and adipocytes [15–18]. Although we know there are various subtypes of CAFs, the precise origin of CAFs remains unclear. ApCAFs is a new subtype of CAFs that expresses major histocompatibility complex class II (MHC II) molecules with a potential role in tumor immunity regulation [19]. The notable increase in T cell counts within squamous cell carcinoma tissues of the head and neck underscores the potential significance of apCAFs in the development of head and neck squamous cell carcinoma [20].

Myeloid cells, which include macrophages, dendritic cells, monocytes and granulocytes, constitute a significant portion of the TME and play interesting roles in immunomodulation, angiogenesis and efferocytosis, thereby influencing tumor progression and metastasis [21]. A study has demonstrated that an increase of CAFs can lead to the infiltration of myeloid lineage cells, such as macrophages, neutrophils, mast cells and eosinophils [22]. CAFs can modulate myeloid-derived suppressor cells (MDSCs) to enhance the stemness of intrahepatic cholangiocarcinoma through the 5-lipoxygenase pathway [23]. Conversely, an increase of MDSCs can elevate the population of CAFs [24]. These data suggest that interactions of myeloid lineage cells and CAFs in the TME mutually contribute to tumorigenesis. Several studies have indicated a significant correlation between myeloid cells and CAFs levels in lung carcinoma and neuroblastoma, which CAFs with positive myeloid cell markers relate with poor prognosis in the HNSCC [25–27]. It is also known that tumor-associated macrophages display unique transcriptional profiles distinct from monocytes and tissue-resident macrophages [28, 29]. Based on the distinctive transcriptional profiles of myeloid cells in the TME, there are a positive correlation between myeloid cells and CAFs as well as higher expression of the key myeloid cell marker MHCII in CAFs [30].

Therefore, understandings of apCAF origin and functions in the HNSCC are critically useful to develop effective therapeutic treatments. Herein, we hypothesized that apCAFs could be differentiated from myeloid cells in the HNSCC. Indeed, we identified apCAFs in the HNSCC by analyzing single-cell sequences from both human and mouse models of the HNSCC and immunohistochemical staining. Further, data from pseudotime analyses and transgenic mouse lineage tracing model uncovered that apCAFs could be differentiated from myeloid in the HNSCC. Also, we unveiled that myeloid cells transdifferentiation was mediated by tumor cell-secreted MIF through the JAK/STAT3 signaling pathway. Interestingly, our data demonstrated that the apCAFs in the HNSCC could regulate T cell proportions and accelerate tumor progression. Our study not only elucidates the mechanisms of the myeloid cell transdifferentiations into apCAFs in the HNSCC, but also reveals how myeloid cell-derived apCAFs affect tumor growth, which potentially provides for novel therapeutic strategies for the HNSCC.

## Materials and methods

### Clinical samples

The collection and utilization of patient samples in this study adhered strictly to the Declaration of Helsinki guidelines. The ethical review committees of Jilin University Stomatology Hospital granted approval for the collection and use of these samples and the approval number is SJDKQ2024033. Human tongue tumor tissues were procured from 6 individuals undergoing surgical treatment for OSCC at the Jilin University Stomatology Hospital. None of the subjects had received any form of treatment prior to surgery. Each tumor sample was used for flow cytometry and image analyses. This study received the ethical endorsement of the Jilin University Stomatology Hospital's ethics committee.

### Animals

All procedures involving animals were conducted in strict accordance with the guidelines set by the Laboratory Animal Ethics Committee of the School of Basic Medical Sciences at Jilin University, China, and complied with established ethical standards, under license number 2024318. C57BL/6 mice were given free access to food and water, and housed in a specific pathogen-free (SPF) environment. Conditions were carefully controlled to maintain a stable temperature of 22 °C and humidity levels at 55 ± 10%, with a consistent 12-h light–dark cycle. The Lyz2Cre;ROSA-mTmG mice were produced through the crossbreeding of Lyz2-Cre (Cyagen, Santa Clara, CA, USA) and ROSA-mT/mG strains (Jackson Laboratory, Bar Harbor, ME, USA).

### Cell cultures

The THP-1 human monocyte cell line was acquired from the Institute of Biochemistry and Cell Biology at the Chinese Academy of Sciences in Shanghai, China. The human oral squamous cell carcinoma cell line, CAL27 and mouse oral squamous cell carcinoma cell line, SCC7 were generously provided by Shanghai Ninth People's Hospital in Shanghai, China. The HOK cell line, an immortalized normal human oral epithelial keratinocyte line, and the human oral squamous cell carcinoma cell lines, SCC25 and SCC9 were obtained from Hycyte Biotechnology Co. (Suzhou, Jiangsu, China). THP-1 cells were cultured in RPMI 1640 medium (Thermo Fisher Scientific, Grand Island, NY, USA) with 10% fetal bovine serum (FBS). SCC7 cells were cultured in RPMI 1640 medium (Thermo Fisher Scientific) with 10% FBS and 1% penicillin/streptomycin. CAL27, SCC25, SCC9 and HOK cells were grown in H-DMEM (Thermo Fisher Scientific) with 10% FBS and 1% penicillin/streptomycin. All cell lines were maintained at 37 °C in a humidified atmosphere containing 5% CO_2_.

### 4NQO model and lineage tracing

Assays of induction of OSCC and lineage tracing were carried out following previous method [31]. 4-Nitroquinoline N-oxide (4-NQO, Santa Cruz Biotechnology, Dallas, Texas, USA) was dissolved in propylene glycol (Sigma-Aldrich, Chaoyang, Beijing, China) at 5 mg/mL and stored at 4 °C for future use. Six-week-old Lyz2-Cre;ROSA-mTmG mice started to drink 4-NQO water at 40 μg/mL for 10 weeks. Then, the mice were given only water from weeks 10 to14. Mice were closely observed the post-treatment effects.

### Subcutaneous co-transplant model

Female C57BL/6N mice (8 and 10 weeks old) were utilized for the subcutaneous co-transplant model. Cultured SCC7 cells, which were screened for mycoplasma contamination, were rinsed with cold PBS, dissociated using 0.25% trypsin, harvested with DMEM containing 10% FBS, centrifugated at 300 g for 3 min, and cell pellet was washed twice with cold PBS. Concurrently, apCAFs were isolated from a digested single-cell suspension of mouse tumor tissue using magnetic beads. SCC7 tumor cells alone at 9 × 10^5^ cells/200 µl or plus apCAFs at 3 × 10^4^ cells/200 µl were subcutaneously injected into the right flank of C57BL/6 mice (*n* = 6/group). For in vivo inhibition experiments, SCC7 cells at 2 × 10^6^/200 µl were inoculated into the backs of mice (*n* = 6/group), and started to get MIF antagonist 4-Iodo-6-phenylpyrimidine (4-IPP, MedChemExpress, Monmouth Junction, NJ, USA) at 5 mg/kg or p-STAT3 inhibitor stattic (MedChemExpress) at 10 mg/kg by intraperitoneal (IP) injection, then injected every 2 days for 15 days. The volume of the tumors was measured calculated using the formula V = 1/2 × length × width^2^.

### Single cell RNA-seq data analyses

Single cell RNA sequence data of human HNSCC were acquired from the GSE103322 dataset [32]. Single cell RNA sequence data of 4NQO-induced OSCC from mouse tongue were obtained from the GSE164817 dataset [33]. Single cell RNA sequence data of human normal oral mucosa were from the GSE164241 dataset [34]. Single-cell sequence data from normal and cancerous groups were harmonized using the Harmony algorithm. Then, cell clusters were annotated based on the significant differential expression of genes across the clusters, identified using the FindAllMarkers function in the Seurat package. Interaction relationships and their strengths among the cell clusters were deduced using the CellChat (version 1.6.1). Furthermore, cell differentiation trajectory analysis was performed utilizing the Monocle (version 2.26.0), which took into account the kinetics of cell gene expression.

### Immunofluorescent staining

Tissues were fixed in 4% paraformaldehyde (PFA), dehydrated through a graded series of alcohols, then embedded in paraffin. Sections of 4 µm thickness were dewaxed to water through a standard protocol. Antigen retrieval was achieved by microwave heating in Tris–EDTA buffer, followed by blocking in 5% BSA in PBS. Subsequently, both human and mouse tissue sections were incubated with primary antibodies, anti-human CD74-APC (Biolegend, San Diego, CA, USA), anti-mouse Cd74-AF647 (Biolegend), anti-human PDGFRB-PE (Biolegend), and anti-mouse PDGFRB-PE (Biolegend) at 4 °C overnight. Nuclei were stained with DAPI (Solarbio, Tongzhou, Beijing, China). Multispectral images were captured using confocal microscopy to analyze.

### Flow cytometry

Tumor tissues from mice or humans were freshly harvested and dissociated into single-cell suspensions using both mechanical and enzymatic digestion methods. Specifically, prepare the enzyme mix by adding 2.35 mL of RPMI 1640, 100 µL of Enzyme D, 50 µL of Enzyme R and 12.5 µL of Enzyme A into a gentleMACS C Tube. The gentleMACS C Tube, Enzyme A, Enzyme D and Enzyme R are derived from the Mouse or Human Tumor Dissociation Kit (Miltenyi Biotec, Germany). Next, remove fat, fibrous tissue and necrotic areas from the tumor sample and cut the tumor into small pieces (2–4 mm). Transfer the tissue into the gentleMACS C Tube containing the enzyme mix. Then, use the gentleMACS Dissociator with the programs 37C_m_TDK_1 and 37C_h_TDK_1 for tissue dissociation. After the program is completed, filter the digested tumor tissue through a 70-μm mesh filter, followed by centrifugation and resuspension to obtain single cells from the dissociated tumor. After dissociation, cells were fixed and stained with primary antibodies. The FACS analysis was performed using MACSQuant Analyzer 16 flow cytometer (Miltenyi Biotec, Bergisch Gladbach, Nordrhein-Westfalen, Germany), and data were analyzed using FlowJo software. For flow cytometry, anti-human CD74-APC, anti-mouse CD74-AF647, anti-human PDGFRβ-PE, anti-mouse PDGFRβ, anti-mouse CD8a-APC and anti-mouse CD4-APC were used. All antibodies were obtained from Biolegend.

### RNA extraction and RT-qPCR analysis

Following incubation of THP-1 cells with or without tumor-conditioned medium from HOK, CAL27, SCC9 and SCC25 at different time points, total RNA was extracted from the THP-1 cells using the DNAiso Plus reagent (Takara, Chaoyang, Beijing, China). cDNAs were amplified using a reverse transcription kit (YEASEN, Pudong, Shanghai, China). Quantitative reverse transcription PCR (RT-qPCR) was conducted employing Hieff qPCR SYBR Green Master Mix (YEASEN) on a Biorad Real-Time Fluorescent Quantitative PCR Detection System. The expression levels of genes in each sample were normalized to ACTB mRNA as an internal control, and the 2^−ΔΔCt^ method was applied for data analysis. Experiments were repeated at least three times.

### Western blot analysis

Proteins were extracted with PIPA lysis buffer, and lysates were used to run electrophoresis on a 10% Tris–Glycine PAGE Gel before being transferred onto polyvinylidene fluoride (PVDF) membrane. The membrane underwent was block with TBST buffer plus BSA, incubated with primary antibodies at 4 °C overnight. Primary antibodies, rabbit anti-CD74 (Proteintech), rabbit anti-FAP (ABclonal), rabbit anti-P-Stat3 (Cell Signaling Technology), rabbit anti-Stat3 (Cell Signaling Technology) and mouse anti-Beta Actin (β-actin) (Proteintech) were used in this study. Then, membrane was incubated with horseradish peroxidase-conjugated secondary antibodies, either goat anti-rabbit or goat anti-mouse (Proteintech) for 1 h at room temperature. Detection was achieved using the HPR substrate ECL (Proteintech), and band intensities were quantified with ImageJ software.

### Enzyme‐linked immunosorbent assay (ELISA)

Blood samples were obtained from both healthy and tumor-bearing mice, allowed to be clotted at room temperature for 1 h, and then centrifuged at 1000 g for 15 min to get serum. Supernatants from SCC25, SCC9, CAL27 and HOK cell cultures were collected after 48 h culture. Levels of MIF in these samples were quantitatively measured using either mouse or human MIF ELISA kits (Proteintech).

### Macrophage depletion

Macrophages were depleted in vivo using Clodronate Liposomes (YEASEN) via intraperitoneal injection. Specifically, Clodronate Liposomes and sterile PBS (for injection) were removed from the refrigerator and allowed to warm to room temperature (18 °C). After natural warming, the Clodronate Liposomes were mixed by gently inverting the vial 8–10 times. A 26-gauge needle was attached to a 1 mL syringe and 200 µL of Clodronate Liposomes was drawn into the syringe. Prior to injection, the syringe was inverted 6 times to ensure thorough mixing. The needle was then inserted at a 30-degree angle into the lower-right abdominal area of the mouse. A total of 200 µL of either Clodronate Liposomes (experimental group) or PBS (control group) was administered. Treatment began one day prior to tumor inoculation and was repeated every two days for a total of 14 days.

### Transfection

On the day before transfection, 2 × 10^5^ SCC7 cells were seeded in a 6-well plate and cultured with 2 mL of DMEM containing FBS and antibiotics. After 24 h, 80 pmol of siRNA (GenePharma) was added to 200 µL of serum-free DMEM and mixed thoroughly. Lipofectamine reagent (GenePharma) was then diluted by adding 4 µL of Lipofectamine to 200 µL of serum-free DMEM, gently mixed and incubated at room temperature for 5 min. The siRNA and Lipofectamine mixtures were combined and incubated at room temperature for an additional 20 min. The resulting siRNA/Lipofectamine complex (400 µL) was added to the 6-well plate, and the plate was gently shaken back and forth. After 24 h of incubation at 37 °C in a CO_2_ incubator, subsequent assays were performed. The siRNA with the highest knockdown efficiency was used for large-scale experiments and tumor inoculation.

### Statistical analysis

All experiments were repeated three times. Statistical analyses, unpaired t-tests were conducted using GraphPad Prism version 9.0 software. Analysis of TCGA data was conducted through the Tumor Immune Estimation Resource 2.0 (TIMER 2.0) (http://timer.cistrome.org). Gene Expression Profiling Interactive Analysis (GEPIA) platform was available at http://gepia.cancer-pku.cn/index.html. Data are presented as mean ± SD or mean ± SEM. Results were deemed statistically significant if *p* < 0.05. *: *p* < 0.05, **: *p* < 0.01 and ***: *p* < 0.001.

## Results

### ApCAFs in human HNSCC

**Fig. 1:**
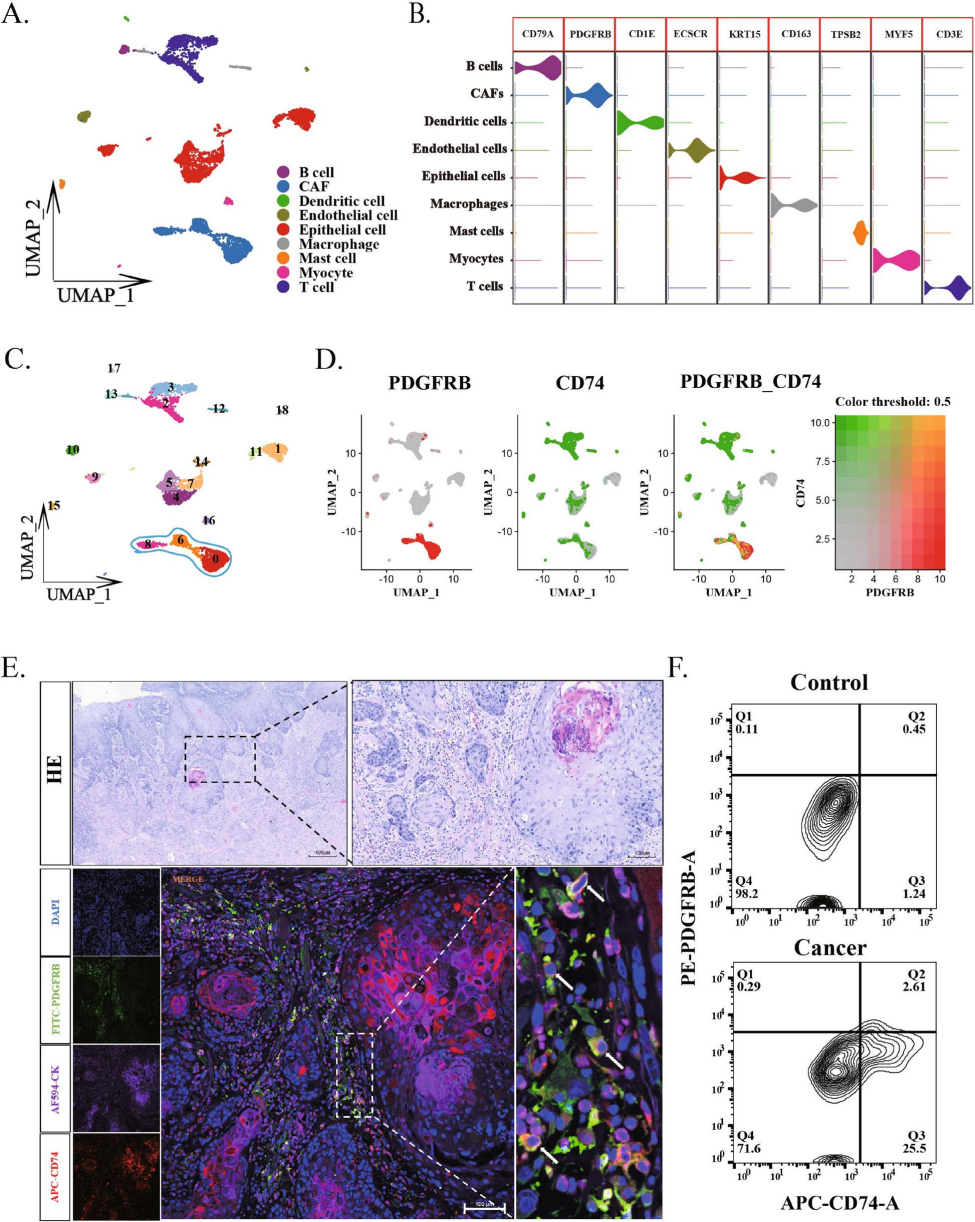
Human oral squamous cell carcinoma cell populations contain PDGFRB^+^CD74^+^ cells. **A** UMAP dimensionality reduction and visualization results show the entire oral squamous carcinoma cell population is divided into 9 cell clusters. **B** Violin plots display the expression levels of highly expressed marker genes across all cell clusters among the nine identified cell clusters. **C** UMAP dimensionality reduction and visualization results further detail the division of the 9 cell clusters into 19 cell subpopulations, with the blue curves highlighting 3 subpopulations of fibroblasts. **D** Feature plots demonstrate the presence of a CAFs cell population co-expressing the genes CD74 and PDGFRB. **E** Multicolor immunofluorescence staining of oral squamous carcinoma tissue shows cells co-expressing CD74 and PDGFRB (indicated by white arrows). **F** Flow cytometry results of oral squamous carcinoma cells reveal the proportion of CD74^+^PDGFRB^+^ cells within the carcinoma (Q2 quadrant)

To understand if apCAFs are in the human HNSCC, single-cell transcriptomic data of HNSCC were downloaded from the GEO database. After quality control and dimensionality reduction, the entire cell population was initially classified into 9 different cell clusters based on classical cell type markers, including epithelial cells, T cells, CAFs, endothelial cells, B cells, myocytes, macrophages, adipocytes and dendritic cells (Fig. 1A and B). To explore subpopulations of CAFs, the entire cell population was further divided into 19 cell clusters, among which clusters 0, 6 and 8 represented subpopulations of CAFs (Fig. 1C). To identify suitable markers for apCAFs in HNSCC, we analyzed single-cell data to examine several commonly used markers for fibroblasts and MHC II. Ultimately, we selected cells co-expressing CD74 and PDGFRB as the definitive markers for apCAFs (Supplementary Fig. 3A). By evaluating the expressions of apCAF markers, CD74 and platelet-derived growth factor receptor beta (PDGFRB) in all cell clusters, we could confirm the presence of apCAFs in cell clusters 6 and 8 in the human HNSCC (Fig. 1D). The CD74 is a MHCII marker that is one of main markers of apCAFs, and also is expressed in the major cell population of myeloid cells [35].

To validate this result, immunofluorescence staining was performed, and images from human OSCC demonstrated that the markers of CD74 and PDGFRB were clearly colocalized (Fig. 1F and Supplementary Fig. 1A). Flow cytometric analysis of human OSCC showed that the positive CD74^+^PDGFRB^+^ apCAFs was 2.61% in the OSCC while the positive CD74^+^PDGFRB^+^ apCAFs was only 0.45% in the normal control tissue (Fig. 1G). Collectively, these results reveal the presence of apCAFs in the human HNSCC.

### ApCAFs in mouse HNSCC

**Fig. 2:**
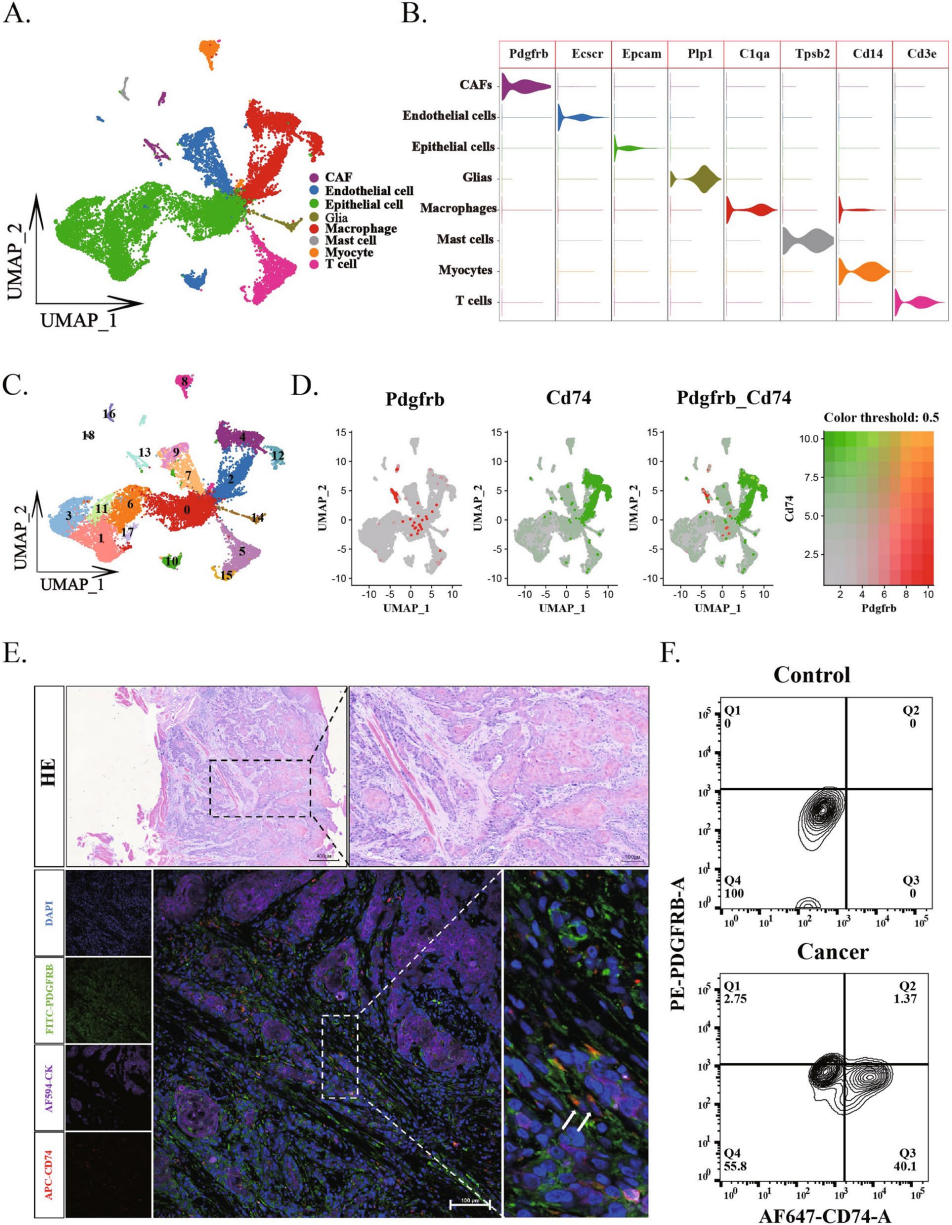
Mouse oral squamous cell carcinoma cell populations contain PDGFRB^+^CD74^+^ cells. **A** UMAP dimensionality reduction and visualization results show the entire oral squamous carcinoma cell population is divided into 8 cell clusters. **B** Violin plots display the expression levels of highly expressed marker genes across all cell clusters among the 8 identified cell clusters. **C** UMAP dimensionality reduction and visualization results further detail the division of the 8 cell clusters into 19 cell subpopulations. **D** Feature plots demonstrate the presence of a CAFs cell population co-expressing the genes CD74 and PDGFRB. **E** Multicolor immunofluorescence staining of oral squamous carcinoma tissue shows cells co-expressing Cd74 and Pdgfrb (indicated by white arrows). **F** Flow cytometry results of oral squamous carcinoma cells reveal the proportion of CD74^+^PDGFRB^+^ cells within the carcinoma (Q2 quadrant)

To further understand origin and functions of apCAFs in the HNSCC, mice model should be used. Therefore, we also needed to know if the apCAFs are in the murine HNSCC. We downloaded single-cell transcriptomic data of murine oral cancer induced by 4NQO from the GEO database. Initially, we categorized the entire cell population into 8 cell clusters using specific cell markers, including epithelial cells, macrophages, endothelial cells, T cells, myocytes, neurons, CAFs and adipocytes (Fig. 2A and B). To identify apCAFs, 19 cell clusters, subpopulations was further categorized (Fig. 2C). Based on expression data of apCAFs markers, CD74 and PDGFRB from all cell clusters, we could determine the presence of apCAFs in the cluster 13 in the murine OSCC (Fig. 2D).

To validate this result, immunofluorescence staining was performed, and images from murine tongue squamous cell carcinoma demonstrated that the markers of CD74 and PDGFRB were clearly colocalized (Fig. 2F and Supplementary Fig. 1B). Additionally, flow cytometric analysis of murine tongue squamous cell carcinoma showed that the positive CD74^+^PDGFRB^+^ apCAFs was 1.37% in the murine tongue squamous cell carcinoma while we could not find any positive CD74^+^PDGFRB^+^ apCAFs in the normal control tissue (Fig. 2G). Clearly, these results indicate the presence of apCAFs in the murine HNSCC.

### Myeloid cells are the main source of apCAFs

As one of the major factors contributing to the heterogeneity of CAFs is their diverse cellular origins, exploring the sources of different subtypes of CAFs could provide novel targets for tumor therapy by modulating the transformation process of CAFs from their source cells. Therefore, we attempted to unravel the origin of apCAFs in the HNSCC.

**Fig. 3:**
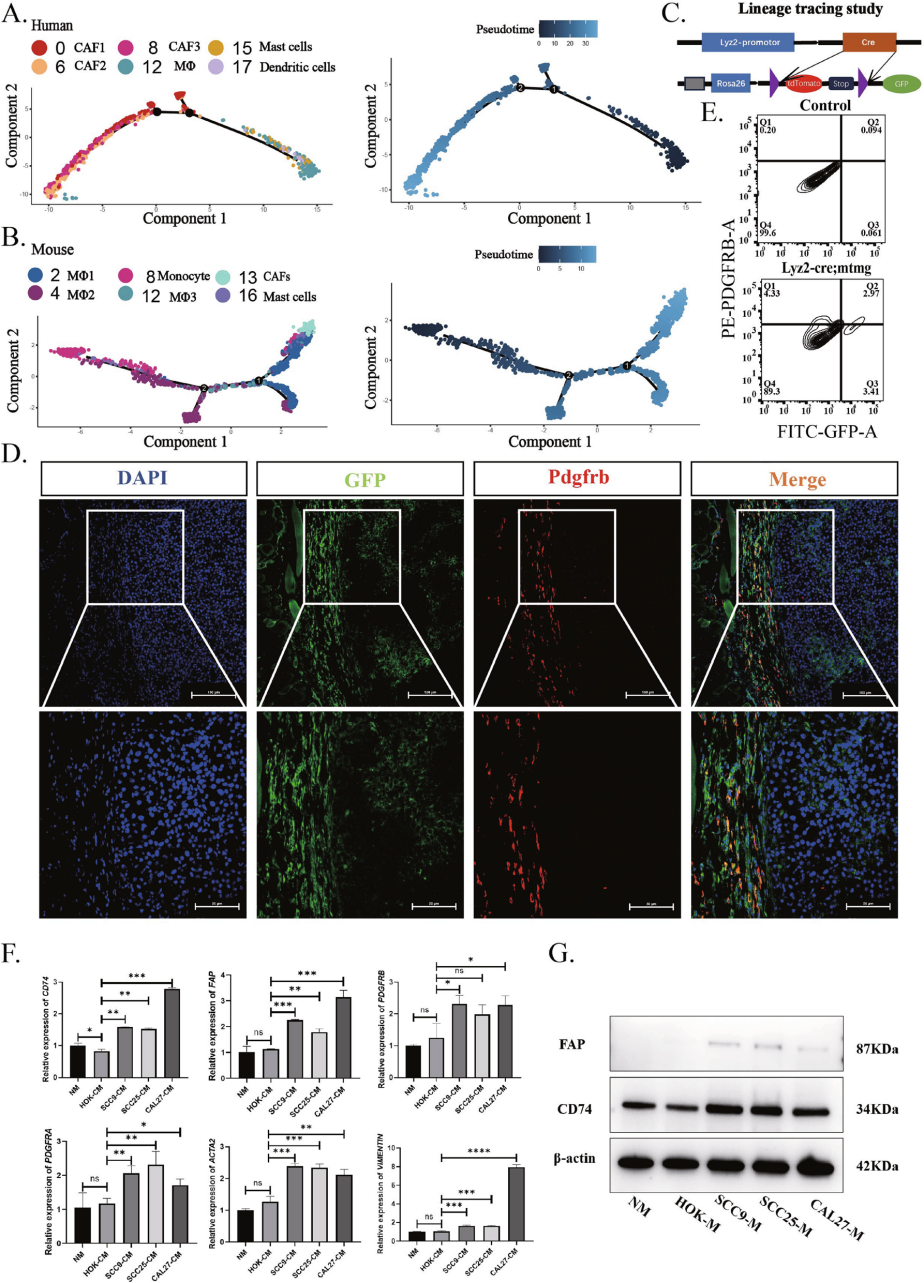
Myeloid cells are capable of differentiating into apCAFs. **A** Pseudotime analysis results of myeloid cells and CAFs in human HNSCC reveal the differentiation trajectory from myeloid cells to CAFs. **B** Pseudotime analysis results of myeloid cells and CAFs in mouse HNSCC show the differentiation trajectory from myeloid cells to CAFs. **C** Illustration of the lineage tracing strategy in Lyz2-Cre/ROSA-mTmG mice, where green fluorescent protein (GFP) is activated following the Cre-mediated excision of a stop codon specific to macrophages, leading to continuous expression of GFP within the myeloid cell lineage. **D** Immunofluorescence staining results of OSCC tissue in Lyz2-Cre/ROSA-mTmG mice show a significant presence of cells co-expressing GFP and PDGFRB protein in the tumor stroma. **E** Flow cytometry results indicate the proportion of cells co-expressing GFP and PDGFRB protein in OSCC of Lyz2-Cre/ROSA-mTmG mice (Q2 quadrant). **F** RT-qPCR analysis represents the increased gene expression levels of *PDGFRA*, *PDGFRB*, *FAP*, *ACTA2*, *VIMENTIN*, and *CD74* in the THP-1 cell following 12 h of stimulation with the CAL 27, SCC25 and SCC9 cell-conditioned medium. **G** Western blot analysis represents the increased protein expression levels of CD74 and FAP in the THP-1 cells after 24 h of stimulation with CAL27 SCC25 and SCC9 cell-conditioned medium. Statistics are shown in mean ± SD (**F**) accessed by the unpaired t test. *, *P* < 0.05; **, *P* < 0.01; ***, *P* < 0.001; ns, nonsignificant, respectively

First, we performed pseudotime analysis on cell populations defined as CAFs and myeloid cells (macrophages, monocytes, dendritic cells, and mast cells) using single-cell sequence data from human and murine HNSCC. Results showed that myeloid cells differentiated earlier than of CAFs in both human and murine HNSCC (Fig. 3A and B), suggesting that myeloid cells could transdifferentiate into apCAFs. Simultaneously, to rule out the possibility that CAFs in the HNSCC originate from epithelial-mesenchymal transition, we performed pseudotime analysis on cell populations defined as CAFs and epithelial cells using single-cell sequence data from human and murine HNSCC. Results showed that the differentiation timeline of CAFs occurred earlier than that of epithelial cells in human HNSCC, and the differentiation timeline of CAFs was partly earlier and partly later than that of epithelial cells in murine HNSCC (Supplementary Fig. 3B). These also suggest that myeloid cells may be one of the primary sources of CAFs in the HNSCC. To further validate pseudotime analysis results in vivo, we generated Lyz2-Cre;ROSA-mTmG mice to trace green positive macrophages (Fig. 3C and Supplementary Fig. 2A, B, C) and used these mice to induce murine OSCC with 4NQO (Supplementary Fig. 2A), then identified myeloid cells (green) and CAFs (red) by immunofluorescence staining in the murine OSCC. Results of images from the immunofluorescence staining clearly showed co-localization of green and red fluorescence (yellow) (Fig. 3D). Further flow cytometry analysis revealed that GFP^+^ and PDGFRB^+^ cells increased in the murine OSCC, 2.97% compared to 0.094% in the control group (Fig. 3E). To further validate pseudotime analysis results in vitro, we cultured THP-1 cells with conditioned medium from CAL 27, SCC 25 or SCC 9 cells for 12 h, and evaluated gene expression levels of CAF markers, VIMENTIN, FAP, PDGFRA, PDGFRB, ACTA2 and MHCII marker, CD74. Interestingly, results demonstrated that tumor-conditioned medium significantly upregulated gene expressions of both CAF markers and MHCII marker (Fig. 3F). After cultured for 24 h, proteins of FAP and CD74 were significantly upregulated by the tumor-conditioned medium (Fig. 3G). These indicates that myeloid cells are one of the main sources of apCAFs in the OSCC.

### Macrophages represent the direct and primary source of apCAFs

**Fig. 4:**
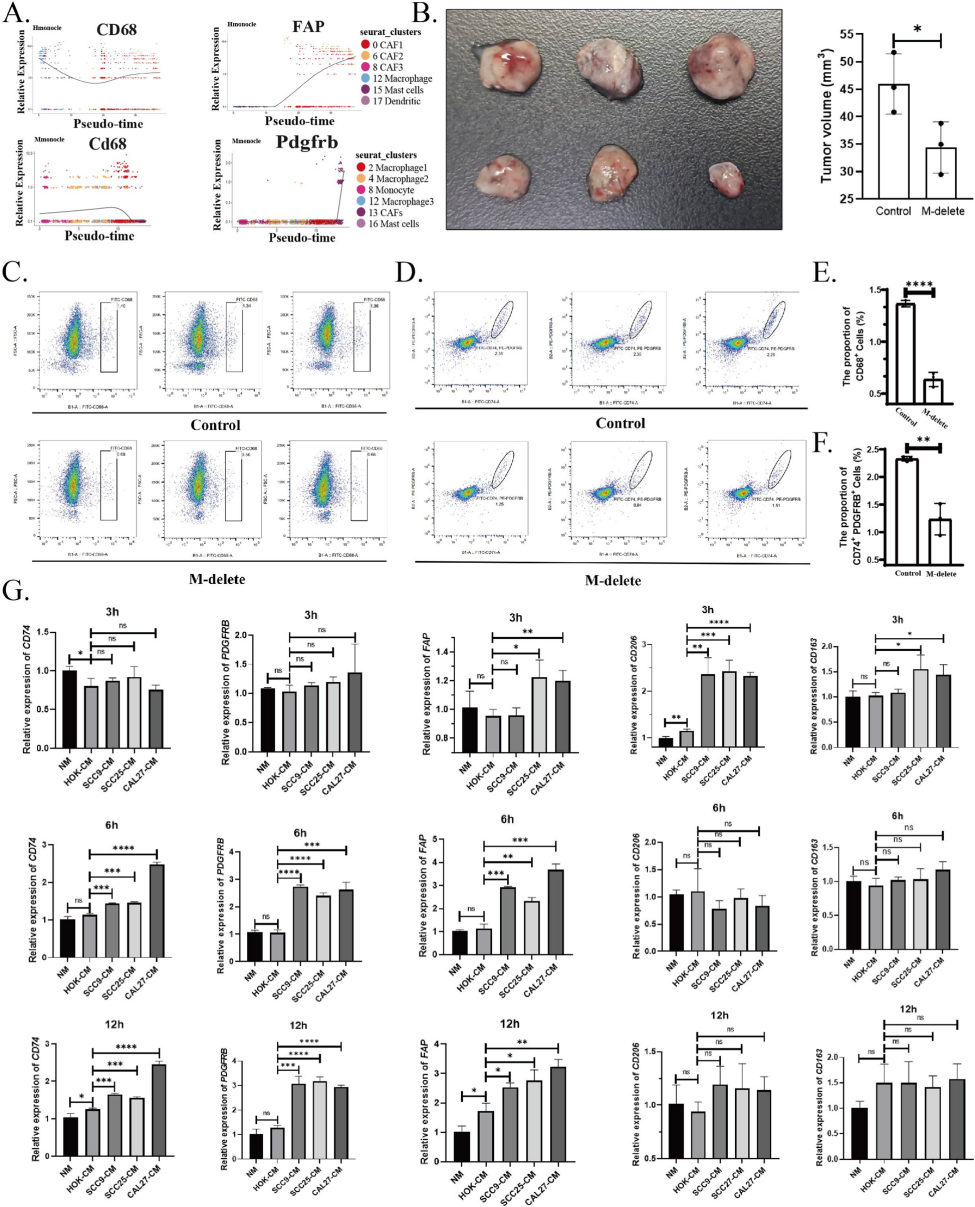
ApCAFs primarily originate from macrophages. **A** Pseudotime analysis of myeloid cells and CAFs based on the expression levels of CD68 and FAP in single-cell sequencing data from HNSCC patients and mice. **B** Gross images and quantitative results showing tumor volume changes in mice after macrophage depletion in vivo. **C** and **E** Flow cytometry dot plots and quantitative results showing changes in the number of CD68^+^ cells after macrophage depletion. **D** and **F** Flow cytometry dot plots and quantitative results showing changes in the number of CD74^+^PDGFRB^+^ cells after macrophage depletion. **G** Expression changes of macrophage and apCAF-related markers after culturing THP1 cells in the culture media of oral squamous carcinoma epithelial cells and normal oral epithelial cells for 3 h, 6 h, and 12 h. Statistics are shown in mean ± SD (**F**) accessed by the unpaired t test. *,* P* < 0.05; **, *P* < 0.01; ***, *P* < 0.001; ns, nonsignificant, respectively

After confirming that myeloid cells are the primary source of apCAFs, we aimed to further investigate which specific subset of myeloid cells predominantly contributes to their generation. To this end, we employed single-cell pseudotime trajectory analysis to examine the dynamic expression of macrophage markers and CAF markers during cellular transformation. Our findings revealed that, in single-cell sequencing data from patients with HNSCC, the expression of CD68 initially decreased and subsequently increased, whereas FAP expression gradually increased. This suggests that macrophages are likely the primary source of apCAFs. However, in single-cell sequencing data from mouse models, we observed that CD68 expression decreased and then increased, while Pdgfrb expression exhibited a direct decrease in CAFs (Fig. 4A). This discrepancy may be attributed to the limited number of CAFs in the single-cell sequencing data from mouse HNSCC, which impeded the differentiation of distinct subpopulations due to data quality limitations. To address this, we performed macrophage depletion in mice and established a tumor model to monitor changes in apCAF numbers. The results showed a significant reduction in tumor volume in the macrophage-depleted group compared to the control group (Fig. 4B). Flow cytometry analysis of CD68^+^ cells revealed a marked reduction in macrophages in the depletion group relative to the control (Fig. 4C and E), confirming the successful depletion of the majority of macrophages in vivo. Furthermore, the number of CD74^+^PDGFRB^+^ and FAP^+^ cells was significantly decreased in the macrophage-depleted group compared to the control (Fig. 4D, F and Supplementary Fig. 4B and E). In vitro, we cultured the THP1 cell line in conditioned media from three OSCC epithelial cell lines and a normal oral epithelial cell line, and assessed apCAF and macrophage-related marker expression at various time points. We found that, compared to the control, 3 h of stimulation resulted in minimal changes in apCAF markers but a significant increase in M2 macrophage markers, with a moderate but observable increase in M1 markers. At 6 and 12 h, there was an increase in apCAF markers and a decrease in macrophage marker expression (Fig. 4G). These results suggest that tumor cell-conditioned media initially prompts THP1 cells to transform into macrophages and then into apCAFs. We also found that MCSF treatment did not induce changes in the gene expression of apCAF markers (Fig. Supplementary Fig. 4A). In conclusion, our findings demonstrate that macrophages within myeloid cells are the primary and direct contributors to the origin of apCAFs.

### Interactions between tumor cells and myeloid cells via the secretion of MIF

**Fig. 5:**
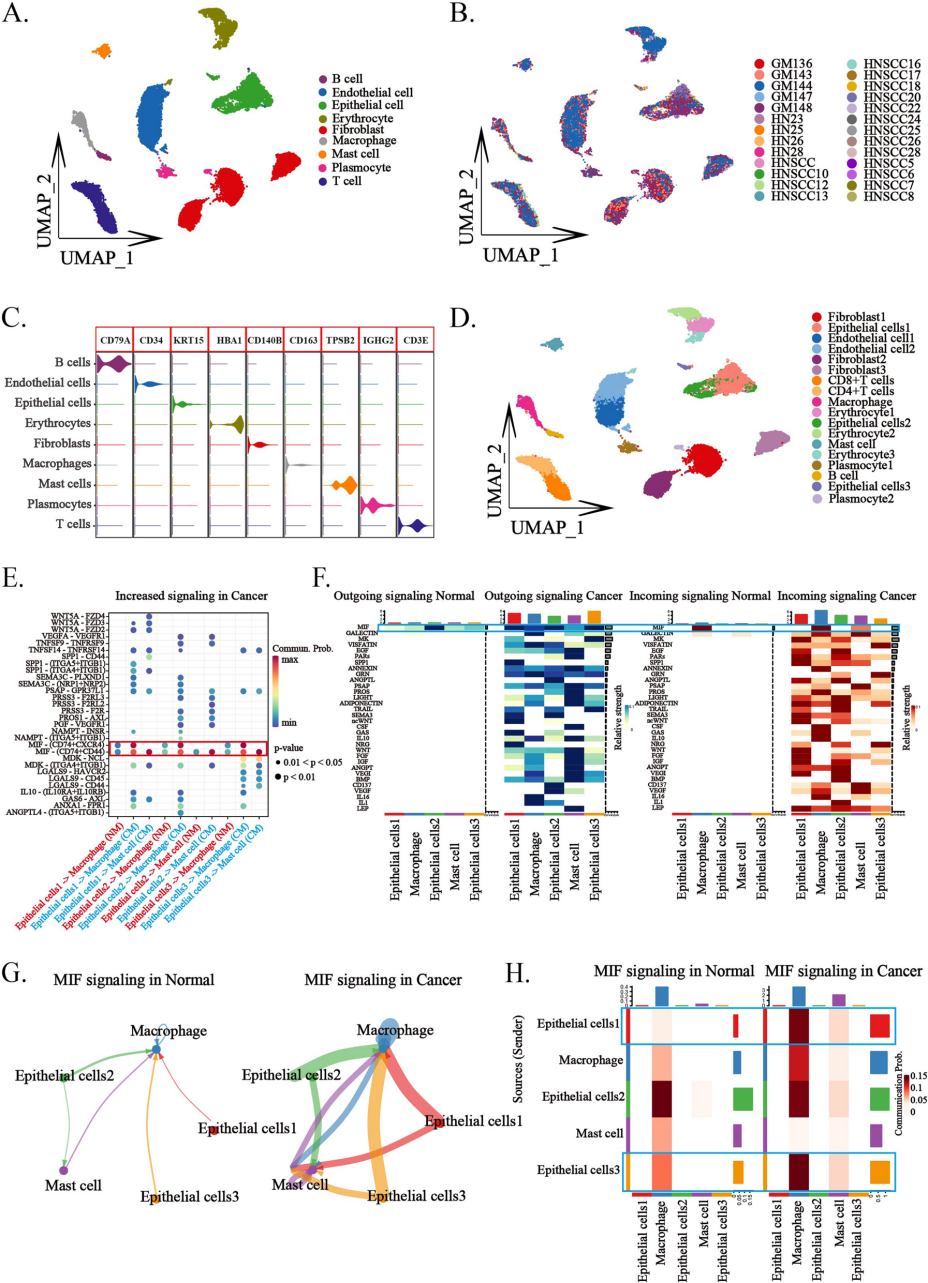
Interaction between tumor cells and myeloid cells via MIF signaling. **A** UMAP dimensionality reduction and visualization categorize the cell populations of the entire oral tumor mucosa and normal oral mucosa into 9 cell clusters. **B** UMAP dimensionality reduction and visualization display the sample origins of all single-cell data and the distribution of cells from each sample across the 9 cell clusters. **C** Violin plots show the expression levels of highly expressed marker genes across all cell clusters within the 9 identified clusters. **D** UMAP dimensionality reduction and visualization results further divide the 9 cell clusters into 18 cell subpopulations. **E** Dot plot highlights enhance interaction signals between epithelial cells and myeloid cells within tumor tissue, predominantly mediated by MIF-CD74. **F** Heatmap illustrates the intensified emission of MIF signals from epithelial cells in tumor tissue and the enhanced reception of MIF signals by myeloid cells within the same context. **G** Shell plots describe the intensity of MIF signaling in tumor groups versus normal groups, emphasizing the increased strength of MIF signaling from epithelial cells to myeloid cells in tumor groups. **H** Heatmap details the intensity of MIF signaling in tumor and normal groups, underscoring the amplified MIF signal strength from epithelial cells to myeloid cells in tumor groups

After identifying myeloid cells as a primary source of apCAFs, next we wanted to elucidate if there are factors that promote myeloid transdifferentiation into apCAFs. Single-cell transcriptomic sequencing data of human normal oral mucosa from the GEO database were used, and integrated these data with single-cell sequencing data from human HNSCC utilizing the Harmony algorithm. The merged dataset comprised samples from 5 human gingival mucosa and 21 HNSCC samples (Fig. 5B). Subsequent quality control and dimensionality reduction segregated the cells into 9 distinct clusters, which were characterized using classical cell markers as epithelial cells, endothelial cells, fibroblasts, erythrocytes, plasma cells, T cells, mast cells, macrophages and B cells (Fig. 5A and C). These clusters were further subdivided into 18 subclusters, and epithelial cell cluster comprised three subclusters (Fig. 5D). Next, epithelial and myeloid cells (macrophages and mast cells) were extracted from the integrated data for intercellular interaction analysis using the CellChat R package. Receptor-ligand interaction analysis revealed a marked enhancement in MIF-CD74 receptor-ligand interaction between epithelial cells and myeloid cells in tumor tissue compared to normal mucosal (Fig. 5E). Differential analysis of incoming and outgoing signals between myeloid and epithelial cells in normal and tumor tissues showed amplified MIF signaling from tumor epithelial cells and enhanced MIF reception in myeloid cells (Fig. 5F). Consequently, we specifically analyzed the MIF signaling between epithelial and myeloid cells. Signaling flow and network differences highlighted a significant augmentation of MIF signaling between epithelial cells and myeloid cells in tumor tissue in contrast to that in the normal mucosal (Fig. 5G and H). These analyses of single-cell sequencing data suggest that tumor cells predominantly interact with myeloid cells through the secretion of MIF in the human HNSCC.

### Knockdown of MIF reduces the number of apCAFs

**Fig. 6:**
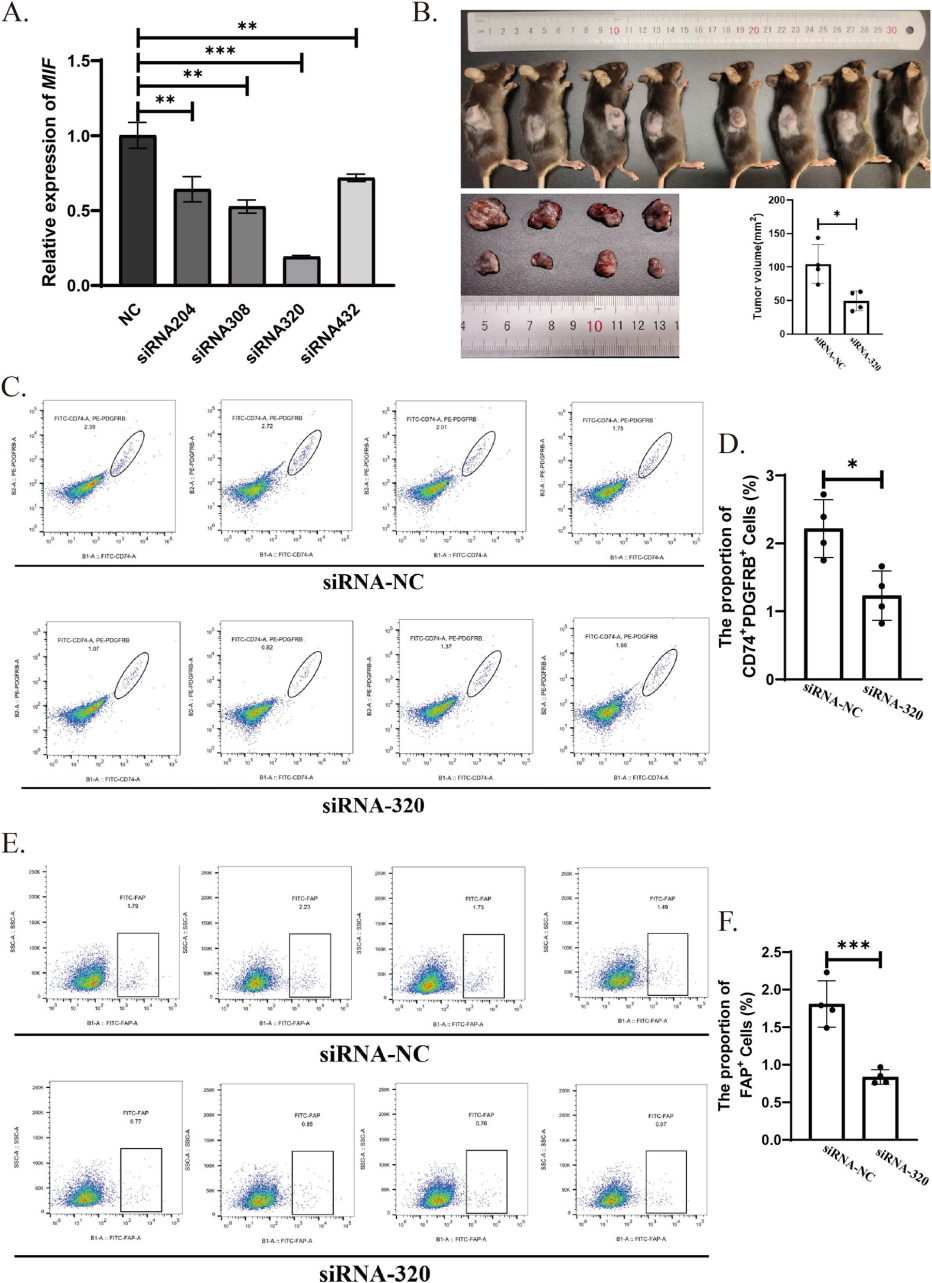
Knockdown of MIF in SCC7 cells results in a decrease in apCAF numbers. **A** Changes in MIF mRNA expression levels in SCC7 cell lines 24 h after treatment with different siRNAs. **B** Gross images and quantitative results showing tumor volume changes 14 days after inoculation of SCC7 cell lines treated with siRNA-320 or siRNA-NC. **C** and **D** Flow cytometry dot plots and quantitative results showing changes in the number of CD74^+^PDGFRB^+^ cells in tumor tissue from both groups of mice. **E** and **F** Flow cytometry dot plots and quantitative results showing changes in the number of FAP^+^ cells in tumor tissue from both groups of mice. Statistics are shown in mean ± SD (**F**) accessed by the unpaired t test. *, *P* < 0.05; **, *P* < 0.01; ***, *P* < 0.001; ns, nonsignificant, respectively

To investigate the role of MIF in the transdifferentiation of myeloid cells into apCAFs, we employed siRNA to knock down the expression of MIF in the SCC7 cell line. Initially, we selected siRNA320, which demonstrated the highest knockdown efficiency (Fig. 6A). Subsequently, we used siRNA320 to knock down MIF expression in SCC7 cells. Tumors were then established using SCC7 cells with reduced MIF expression. The results showed that, compared to the control group, MIF knockdown in SCC7 cells resulted in a reduction in tumor size, accompanied by a significant decrease in the number of both CD74^+^PDGFRB^+^ and FAP^+^ cells (Fig. 6C, D, E and F). These findings indicate that MIF derived from the SCC7 cell line plays a critical role in the differentiation of myeloid cells into apCAFs.

### MIF induces transformation of myeloid cells into apCAFs through JAK/STAT3 signaling pathway

**Fig. 7:**
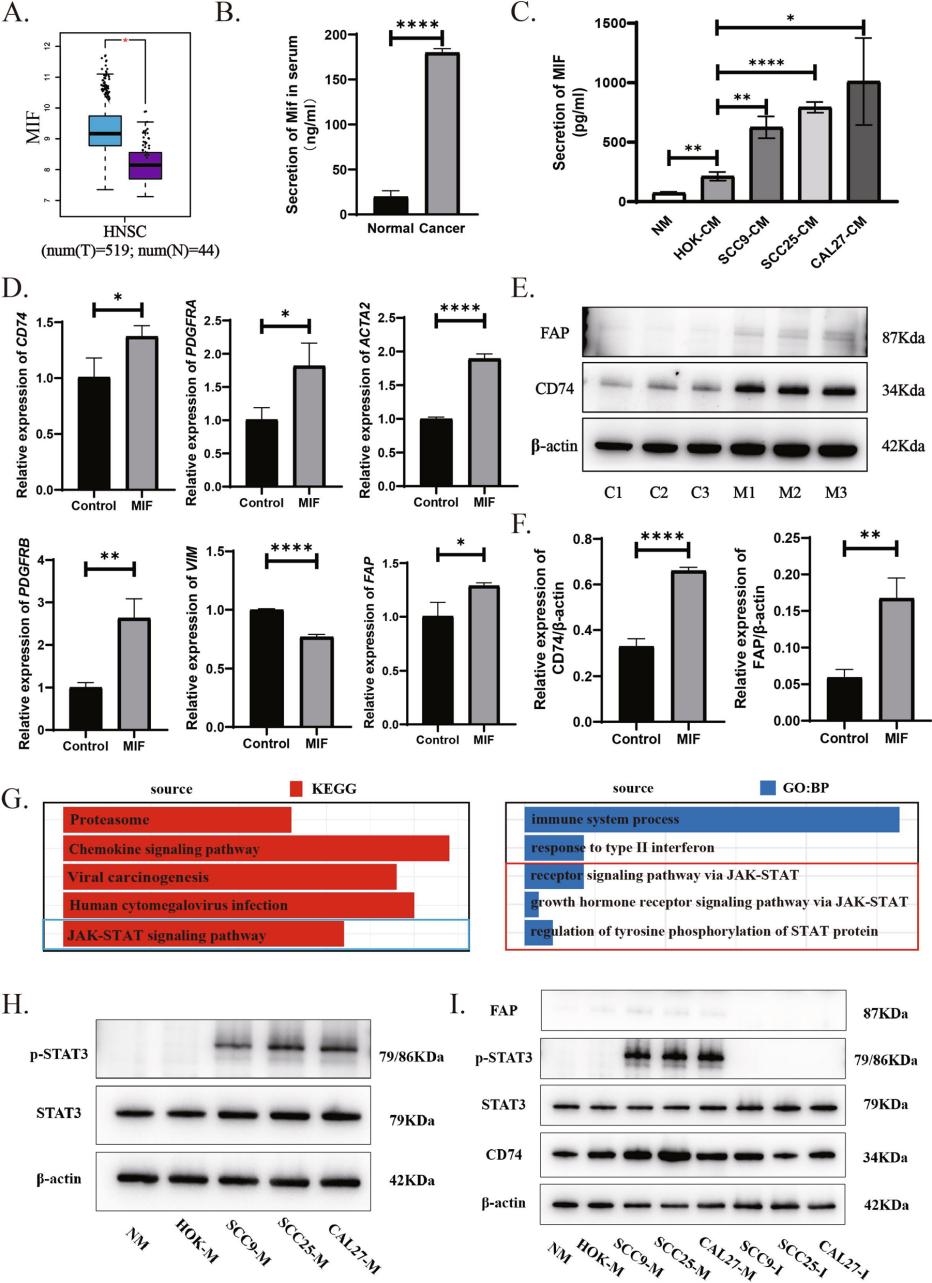
Tumor-derived MIF promotes myeloid cell phenotypic transition through the JAK/STAT signaling pathway.** A** *MIF* expression results from HNSCC bulk RNA sequencing data from the TCGA database are presented, showing range and mean ± SEM. Statistical analysis was conducted using the Wilcoxon test. **B** Protein expression levels of MIF in serum from normal mice versus mice with OSCC. **C** Protein expression levels of MIF in conditioned media from normal oral epithelium and OSCC epithelial cells. **D** Changes in mRNA expression levels of *CD74*, *PDGFRA*, *ACTA2*, *PDGFRB*, *VIMENTIN*, and *FAP* in THP-1 cells treated with or without MIF. **E** and **F** Changes and quantitative bar graphs of protein expression levels of FAP and CD74 in THP-1 cells treated with or without MIF. **G** A bar chart presents the results of GO and KEGG enrichment analysis of differentially expressed genes in myeloid cells between HNSCC and normal oral mucosa. **H** Changes of p-STAT3 protein expression levels in THP-1 cells treated with conditioned media from normal oral epithelium and OSCC epithelial cells. **I** Changes in protein expression levels of p-STAT3, CD74 and FAP in THP-1 cells treated with conditioned media from normal oral epithelium and OSCC epithelial cells, and treated with the MIF inhibitor HY13818. Statistics are shown in mean ± SD (**B**, **C**, **D**, and **F**) accessed by the unpaired t test. *, *P* < 0.05; **, *P* < 0.01; ***, *P* < 0.001; ns, nonsignificant, respectively

To further understand if MIF also mediate the transdifferentiation of myeloid cells into apCAFs, we analyzed expression levels of the MIF gene in human HNSCC and normal tissues using the TCGA database, and uncovered significant upregulation in tumor compared to normal tissues (Fig. 7A). In the meantime, our data from Elisa assays demonstrated that protein expression levels of MIF were increased in OSCC cell lines compared to normal oral mucosa epithelial cell lines in vitro (Fig. 7C). Additionally, MIF protein was also increased in the serum from squamous carcinoma mice compared to the serum from normal mice in vivo (Fig. 7B).

Subsequently, to explore if MIF can induce the transdifferentiation of myeloid cells into apCAFs, THP-1 cells were cultured with human recombinant MIF for 12 h, then gene expression of CAFs markers, VIMENTIN, FAP, PDGFRA, PDGFRB and ACTA2, as well as MHCII marker CD74 were evaluated. Results showed that all these genes were significantly upregulated post-treatment with MIF (Fig. 7D). Also, proteins of FAP and CD74 were significantly increased from the same cultures with human recombinant MIF for 24 h (Fig. 7E and F). These findings suggest that MIF can induce the transdifferentiation of myeloid cells into apCAFs.

To understand mechanism of MIF effects on the transdifferentiation of myeloid cells into apCAFs, differential gene analysis was performed on myeloid cells in the HNSCC and normal tissues from the merged single-cell data, and followed by KEGG and GO enrichment analyses. Results indicated that the differential genes in myeloid cells of human HNSCC were mainly enriched in the JAK/STAT3 signaling pathway (Fig. 7G). To validate this enrichment analysis result, protein, which extracted from THP-1 cells cultured with conditioned medium from CAL 27, SCC 25 or SCC 9 cells for 24 h, was used to run Western Blotting in order to evaluate p-STAT3 level. Results showed that the p-STAT3 was dramatically increased by MIF (Fig. 7H). Lastly, inhibitor of JAK/STAT3 signaling pathway, HY13818 could significantly suppressed phosphorylation of STAT3 protein and reduced the protein levels of CD74 and FAP (Fig. 7I). Our data clearly demonstrated that MIF induces the transdifferentiation of myeloid cells into apCAFs via the JAK/STAT3 signaling pathway.

### ApCAFs modulate the T cell ratio to facilitate HNSCC progression

**Fig. 8:**
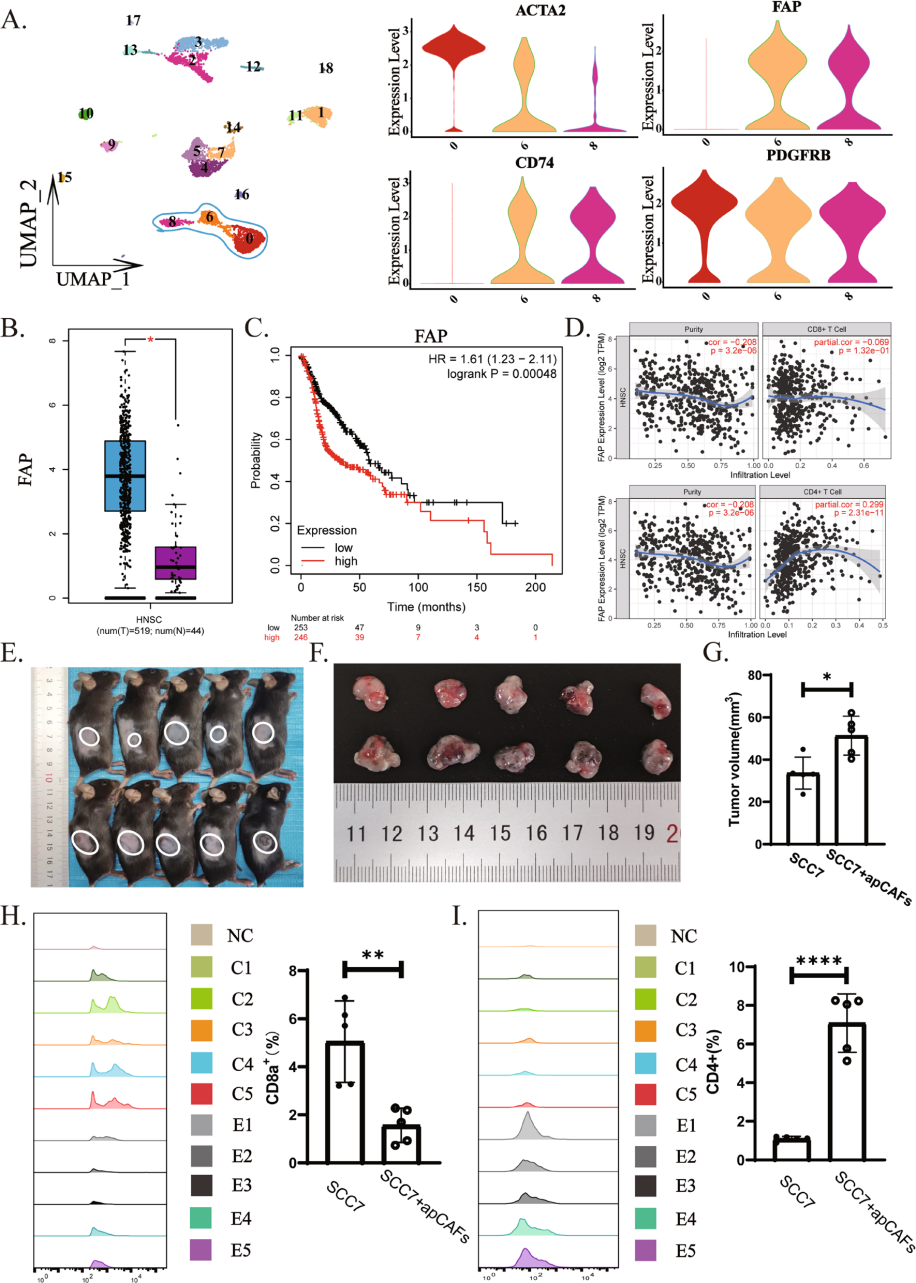
ApCAFs regulate T cell proportions to promote tumor progression.** A** UMAP dimensionality reduction and visualization categorize HNSCC cells into 19 cell subgroups and Violin plots show the gene expression levels of ACTA2, FAP, CD74 and PDGFRA in cell subgroups 0, 6 and 8. **B** FAP expression results from bulk RNA sequencing data of HNSCC from the TCGA database. Data are presented as range and mean ± SEM, analyzed by the Wilcoxon test. **C** Survival analysis of patients with HNSCC reveals that patients with high FAP expression have a lower overall survival rate (log-rank test, *p* = 0.00048). **D** Correlation analysis between FAP expression and CD4^+^ T cells and CD8^+^ T cells show a positive correlation with CD4^+^ T cell infiltration and a negative correlation with CD8^+^ T cell counts. **E**, **F** and **G** In vivo, apCAFs promote tumor growth. **H** Flow cytometry results demonstrate that apCAFs decrease the number of CD8^+^ T cells. **I** Flow cytometry results show that apCAFs increase the number of CD4^+^ T cells. Statistics are shown in mean ± SD (**G**, **H** and **I**) accessed by the unpaired t test. *, *P* < 0.05; **, *P* < 0.01; ***, *P* < 0.001; ns, nonsignificant, respectively

Next, we are interested in exploring the roles of apCAFs in the HNSCC progression. First, we used violin plots to evaluate expressions of CAFs markers in HNSCC single-cell transcriptomic data, and identify that clusters 0 and a proportion of clusters 6 mainly comprised ACTA2^+^ myofibroblasts while cluster 8 and a part of cluster 6 were predominantly apCAFs. We also observed that FAP^+^ CAFs were essentially CD74^+^PDGFRB^+^ cells, i.e., apCAFs. Thus, we utilized the FAP^+^ cell population to represent apCAFs to investigate relationships between apCAFs and tumor progression and prognosis (Fig. 8A and Supplementary Fig. 3A). Subsequently, we compared the expression of FAP gene in the human HNSCC to that in healthy individuals leveraging the TCGA database. Result showed that FAP gene expression significantly increased in the human HNSCC (Fig. 8B). Further database analysis correlating FAP gene expression with the prognosis of head and neck carcinoma patients revealed that higher expression of FAP was associated with poorer prognosis (Fig. 8C). Additionally, we performed survival analysis using the myofibroblast marker ACTA2 in TCGA, comparing normal and tumor tissues. The results revealed no statistically significant differences (Supplementary Fig. 3C and D). Analyzing the relationship between FAP gene expression and T cell infiltration in the human HNSCC found that FAP gene expression negatively correlated with CD8^+^ T cell infiltration and positively with CD4^+^ T cell infiltration (Fig. 8D). These results preliminarily indicate that the apCAFs facilitate HNSCC progression.

To validate the effects of apCAFs on T cells in the HNSCC, apCAFs from mouse OSCC were magnetically sorted, and co-inoculated them with the mouse OSCC cell line, SCC7 cells into the dorsa of mice. After 15 days, we observed that size of tumors was significantly larger in the co-inoculation group than that of the SCC7 alone (Fig. 8E, F, G and Supplementary Fig. 1C, 5A and C). Finally, flow cytometry analyses demonstrated that CD8^+^ T cells decreased, and CD4^+^ T cells increased in the tumors from co-inoculation group while the SCC7 alone group was opposite (Fig. 8H and I). Furthermore, the results showed a significant increase in the number of apCAFs in the co-inoculation group compared to the control group (Supplementary Fig. 5B and D). Moreover, following macrophage depletion, we observed an increase in the number of CD8^+^ T cells, while the number of CD4^+^ T cells decreased (Supplementary Fig. C, D, F and G). Collectively, these findings clearly demonstrate that the apCAFs in the HNSCC regulate the CD4^+^/CD8^+^ T cell ratio, thereby promoting tumor growth.

### MIF and STAT3 are effective therapeutic targets for the HNSCC

**Fig. 9:**
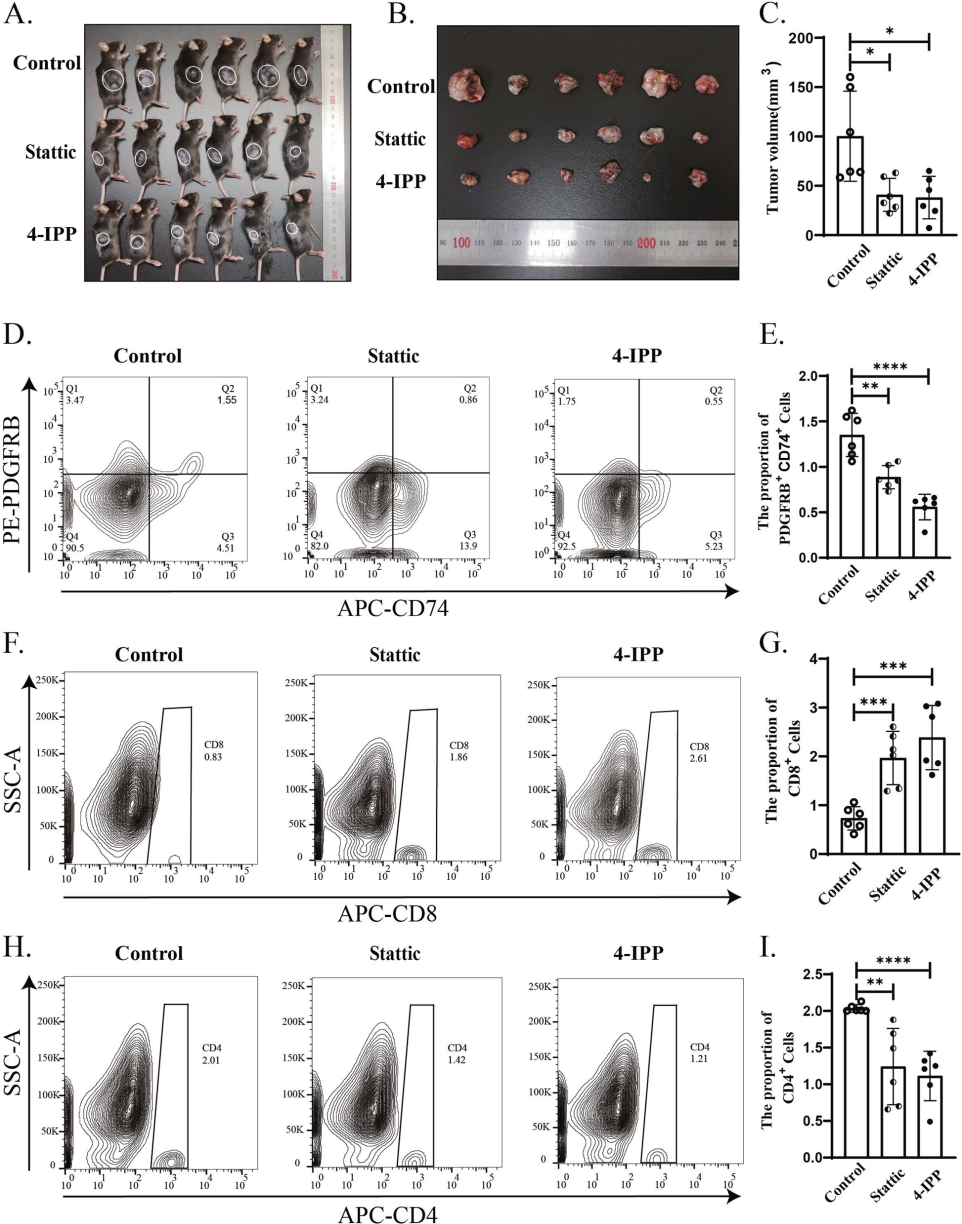
The inhibition of the MIF and p-STAT3 pathways effectively suppresses the growth of OSCC.** A**, **B** and **C** In vivo, inhibition of MIF and p-STAT3 can reduce tumor size. **D** and** E** Flow cytometry results demonstrate that inhibition of MIF and p-STAT3 decrease the number of CD74^+^ PDGFRB^+^ cells. **F** and **G** Flow cytometry results show that inhibition of MIF and p-STAT3 increase the number of CD8^+^ T cells. **H** and **I** Flow cytometry results show that inhibition of MIF and p-STAT3 decrease the number of CD4^+^ T cells. Statistics are shown in mean ± SD (**C**, **E**, **G** and **I**) accessed by the unpaired t test. *, *P *< 0.05; **, *P* < 0.01; ***, *P* < 0.001; ns, nonsignificant, respectively

Most importantly, we want to explore if we can target to inhibit the differentiation of myeloid cells into apCAFs in vivo leading to block tumor growth by regulating the proportion of T cells. 4-IPP is inhibitor of MIF, and stattic is inhibitor of p-STAT3. The mouse OSCC cell line, SCC7 cells were inoculated into the dorsa of mice, and received 4-IPP or static by IP injection. After 15 days post-tumor inoculation, all tumors were collected. Interestingly, we found that tumor volumes from the 4-IPP and stattic injection groups were significantly reduced compared to the control group (Fig. 9A, B, and C). Subsequently, we utilized flow cytometry to assess numbers of apCAFs, CD8^+^ and CD4^+^ T cells from tumors of all three groups. Compared to the control non-treated group, apCAFs were significantly decreased in the 4-IPP or stattic treated group (Fig. 9D and E), and CD4^+^ T cells were decreased (Fig. 9H and I), while CD8^+^ T cells were increased in the 4-IPP or stattic treated group (Fig. 9F and G). These findings further demonstrate that MIF and the phosphorylation of STAT3 signaling pathways mediate the increase of apCAFs. Also, all our data/results indicate that MIF and STAT3 are potential effective therapeutic targets for the HNSCC in the future.

## Discussion

In many cancers, such as pancreatic, lung and breast cancer, CAFs play a crucial roles in the oncogenesis, progression, metastasis, drug resistance and immune evasion of the HNSCC [36]. This is particularly significant as approximately 80% of tumor mass that consists of CAFs in late-stage HNSCC patients [37]. Therefore, investigating origins, functions and underlying mechanisms of CAFs may help to develop novel therapeutic targets for the treatment of HNSCC. In our current study, utilizing single-cell transcriptome sequencing data from public databases, combined with in vitro and in vivo rigorous biological experiments in the lab, we identified the apCAFs, a critical subtype of CAFs in the HNSCC. Further data revealed that myeloid cells are the primary source of the apCAFs in the HNSCC. Interestingly, the MIF secreted by tumor cells is a key factor to induce the transdifferentiation of myeloid cells into apCAFs through the JAK/STAT3 signaling pathway. Our data clearly demonstrate that apCAFs in the HNSCC can promote tumor progression and negatively impact patient prognosis, and also suggest a potential therapeutic strategy for the treatment of the HNSCC by blocking the transformation process of myeloid cells into these CAFs.

CAFs are a crucial heterogeneous cell type within the TME, which involve in various biological processes, such as tumorigenesis, progression, metastasis and drug resistance [38]. CAFs represent a diverse group of cells with significant phenotypic and functional heterogeneity [39]. A recently identified subset within this group, known as apCAFs, with immunomodulatory capabilities [30]. This subset is distinguished by co-expression of both CAFs and antigen-presenting cell (APC) markers. Unlike initially identified myCAFs and iCAFs, apCAFs not only contribute to the synthesis of the extracellular matrix and secretion of inflammatory factors, but also embody the functional attributes of APCs [19]. The apCAFs derive from mesenchymal cells in pancreatic cancer, can induce the expansion of regulatory T cells (Treg cells) leading to an immune-suppressive microenvironment and tumor immune evasion [40]. Through interactions with adaptive immune cells, they play an integral role in anti-tumor immune responses. There are concrete evidences of apCAF presence in pancreatic, lung and breast cancers [40–42]. Their existence in the HNSCC, however, remains to be clarified.

Through single-cell bioinformatics analysis using public available databases, we identified apCAF clusters in human and murine HNSCC by co-expression of special markers of both CAFs and APCs (Figs. 1 and 2). Further, our experimental data from immunofluorescence staining and flow cytometry indeed confirmed that there are apCAFs in the HNSCC. Previous study have categorized CAFs into three subgroups, myofibroblasts, inactive resting fibroblasts and activated fibroblast clusters through TSNE dimensionality reduction of single-cell, but did not recognized the existence of apCAFs in the HNSCC [32]. Our analyses and results of experiments clearly revealed that the apCAFs are in the HNSCC.

It is known that the origin of CAFs is complicated, and has diversity of cellular sources [43]. The CAFs, can be from resident fibroblasts, mesenchymal stem cells (MSCs), adipocytes, adipose-derived MSCs, mesothelial cells, endothelial cells, myeloid cells, pericytes, epithelial cells, hematopoietic stem cells and circulating bone marrow-derived fibrocytes [9]. In vitro studies and bone marrow transplantation indicate that these cells are kind of precursors of CAFs [44]. Notably, there is less detailed information regarding the origin of various CAF subtypes. Recent studies have elucidated the transdifferentiation potential of stromal cells into apCAFs in various cancers, such as mesothelial cell of pancreatic cancer [40]. Interestingly, cancer cells from small cell lung cancer can undergo EMT into apCAFs [41]. Progenitor cells of apCAFs in the HNSCC, however, remain unclear. To explore the origin of apCAFs in the HNSCC, pseudotime analysis and lineage-tracing experiments were employed. Our data from Fig. 3 supported that myeloid cells can transdifferentiate into apCAFs in the HNSCC. Recently, several other study have showed that macrophages can transdifferentiate into myCAFs in Lewis lung carcinoma [45] and, monocytes can transdifferentiate into myCAFs in pancreatic ductal carcinoma [46]. These similar findings not only indicate our result possibility, but also suggest that such transdifferentiation processes might be applicable across various types of cancers.

Several factors have been identified as contributing to the reprogramming of CAFs or the activation of fibroblasts under cancerous conditions. These include epithelial signals, such as interleukin-1 (IL-1), platelet-derived growth factor (PDGF), and transforming growth factor-beta (TGF-β), along with metabolic reprogramming, oxidative stress, stromal signals, microRNAs, epigenetic alterations and other ligands secreted by CAFs [47–52]. Among these, cytokines produced by tumors play a dominant role. For the first time, our data from analyses of intercellular interactions and in vitro experiments clearly demonstrated that squamous cell carcinoma cells of the head and neck mediate the transdifferentiation of myeloid cells into CAFs through the secretion of MIF (Fig. 4). MIF, a homo-trimeric protein, functions as a pleiotropic pro-inflammatory cytokine [53]. MIF involves in the development and progression of various types of cancer, such as gastric cancer, bladder cancer, ovarian cancer and esophageal cancer by interfering cell cycle, inhibiting anti-tumor immunity, promoting tumor cell proliferation and angiogenesis [54]. It has found that tumor cells reshape the heterogeneity of macrophages in the TME by secreting MIF in a soft tissue sarcoma mouse model [55]. In the presence of MIF, myeloid cells exhibit pro-tumor effects. Conversely, knockdown of MIF leads to anti-tumor effects of myeloid cells, which attributes to the reduction of SPP1^+^ macrophages that play roles in extracellular matrix remodeling. Given fibroblasts are the primary cell type involved in the synthesis and secretion of the extracellular matrix, it can be inferred that SPP1^+^ macrophages with matrix remodeling capabilities are likely apCAFs at this juncture. This inference aligns our findings, suggesting a pivotal role of MIF in shaping the TME by influencing cellular phenotypes.

Multiple signaling pathways are implicated in the transdifferentiation of the TME into CAFs, such as the NF-κB signaling pathway, the Smad3 signaling pathway and the JAK/STAT signaling pathway [45]. The transformation of iCAFs can be promoted by IL-1 through the activation of the JAK/STAT signaling pathway in pancreatic ductal adenocarcinoma [56]. Similarly, P63 can regulate IL-1β to induce the formation of iCAFs through the JAK/STAT pathway [57]. Tumor-derived IL-6 promotes the formation of inflammatory CAFs via the JAK/STAT pathway through co-culture of tumor organoids and CAFs [44]. These studies collectively underscore the pivotal roles of the JAK/STAT signaling pathway in the phenotypic transformation of CAFs. Our results also revealed frequent enrichment of genes in the JAK/STAT signaling pathway from analyses of high-expression differential genes of myeloid cells in tumor tissue compared to myeloid cells in normal tissue using KEGG and GO databases (Fig. 7). Further, we confirmed that the JAK/STAT signaling pathway indeed plays a crucial role in mediating the effects of MIF in the HNSCC by Western Blotting using JAK/STAT pathway inhibitors (Fig. 7). These suggest that the feasibility of targeting the JAK/STAT signaling pathway can be used to regulate the formation of CAFs and ultimately treat cancer.

ApCAFs, characterized by their expression of MHCII proteins, exert an immunomodulatory role in various cancers, such as PDAC, breast cancer and lung cancer [40, 41, 58]. Functions and mechanisms of apCAFs, however, are different across tumor types. For instance, in pancreatic cancer, apCAFs facilitate tumorigenesis by inducing the differentiation of naïve T cells into regulatory T cells (Tregs), thereby creating an immunosuppressive microenvironment [40]. In contrast, apCAFs exert tumor-suppressive effects by producing C1q in small cell lung cancer, which binds to the C1qbp on the surface of CD4^+^ effector T cells, preventing their apoptosis [41]. The role and mechanism of apCAFs in the HNSCC remain unclear. Analyses of TCGA data and our data from in vivo experiments have demonstrated that apCAFs in the HNSCC can influence the ratio of CD4^+^/CD8^+^ T cells, ultimately promoting tumor growth (Figs. 6 and 7). Interestingly, our results from in vivo blocking assays using 4-IPP, MIF inhibitor and stattic, p-STAT3 inhibitor clearly demonstrated that sizes of tumor from the 4-IPP and stattic injection groups are significantly reduced compared to the control group (Fig. 7). Data from flow cytometry showed that apCAFs and CD4^+^ T cells were significantly decreased while CD8 + T cells were increased in the 4-IPP or stattic treated group (Fig. 7). Notably, in our exploration of apCAF functions, we observed an overlap between FAP^+^ CAFs and apCAFs. Studies have confirmed that FAP^+^ CAFs possess immunosuppressive functions [59]. In particular, FAP has been targeted in preclinical therapeutic strategies with vaccines, antibodies, and chimeric antigen receptor (CAR) T cells [60]. Similarly, the genetic depletion of FAP1 CAFs, as well as inhibition of CXCR4 by AMD3100, has been shown to increase T cell infiltration and enhance the efficacy of checkpoint inhibitors in PDAC [61]. This indirectly supports our findings and suggests that therapeutic strategies targeting FAP^+^ CAFs may also be applicable to develop treatment for the HNSCC. Finally, our findings indicate that once myeloid cells transdifferentiate into apCAFs, they express higher levels of CD74. The presence of MIF in the tumor microenvironment likely binds to CD74 on apCAFs. Studies suggest that the interaction between MIF and CD74 may modulate the expression of the chemokine CXCL12 in apCAFs through the activation of the Wnt and TGF-β signaling pathways. This, in turn, could recruit MDSCs and naïve T cells, potentially contributing to the increase in CD4^+^ T cells and the decrease in CD8^+^ T cells, which may play a critical role in the development of an immunosuppressive tumor microenvironment [62–64]. This mechanism may partially explain how apCAFs regulate T cell ratio changes in the head and neck squamous cell carcinoma microenvironment.

In conclusion, our study reveals the complex interplay within the TME between tumor cells and myeloid cells. These interactions induce the transdifferentiation of apCAFs, ultimately promoting tumor growth through immunosuppression. Our findings provide useful profound understandings for the heterogeneity and mechanisms of CAFs in the HNSCC. All results suggest that we can use our novel findings to develop the target strategy within the transdifferentiation process of myeloid cells into apCAFs and modulation of apCAFs on T cells to treat the HNSCC.

## Supplementary Information

Supplementary Material 1: Fig. 1. HE staining of tumor tissues from different HNSCC models in both patients and mice. A, Hematoxylin and eosin (H&E) stained histological sections of tumor tissues from patients with OSCC used in this study. B, H&E staining histological sections of OSCC samples induced by 4NQO in mice. C, H&E staining histological sections of murine transplant tumors following inoculation with the mouse OSCC cell line SCC7. Fig. 2. Successfully established a myeloid cell-specific fluorescent reporter mouse and an OSCC model. A, Tissue specimens of normal mouse tongue and 4NQO-induced OSCC in mouse tongue. B, Genotyping results for Lyz2-Cre/ROSA-mTmG mice. C, Fluorescence identification results of spleen in Lyz2-Cre;ROSA-mTmG mice. Fig. 3. Single-cell data and TCGA database analysis results. A, the expression levels of different fibroblast-associated markers (COL3A1, COL1A1, PDGFRB, FAP) and MHC class II-related markers (HLA-DPB1, HLA-DRA, HLA-DPA1, CD74) across three CAFs subpopulations in HNSCC. B, Pseudotime analysis results of epithelial cells and CAFs in human and mouse HNSCC. C, ACTA2 expression results from bulk RNA sequencing data of HNSCC from the TCGA database. Data are presented as range and mean ± SEM, analyzed by the Wilcoxon test. D, Survival analysis of patients with HNSCC shows that high ACTA2 expression is not associated with overall survival rates. Fig. 4. The macrophage clearance leads to an increase in the CD8^+^ T/CD4^+^ T ratio. A, Expression changes of macrophage markers and apCAF-related markers in THP1 cells after stimulation with different concentrations of MCSF for 6 h. B and E, Flow cytometry dot plots and quantitative results showing the changes in the number of FAP^+^ cells after macrophage depletion. C and F, Flow cytometry dot plots and quantitative results showing the changes in the number of CD4^+^ T cells following macrophage depletion. D and G, Flow cytometry dot plots and quantitative results showing the changes in the number of CD8^+^ T cells after macrophage depletion. Statistics are shown in mean ± SD (F) accessed by the unpaired t test. *, *P* < 0.05; **, *P* < 0.01; ***, *P* < 0.001; ns, nonsignificant, respectively. Fig. 5. Co-inoculation of apCAFs with SCC7 results in an increased number of apCAFs in the tumor tissue. A, Gross morphology and tumor tissue images of mice 14 days after inoculation with SCC7 alone or co-inoculation of SCC7 and apCAFs. B, Flow cytometry dot plots showing the changes in the number of FAP^+^ cells in tumor tissue 14 days after inoculation with SCC7 alone or co-inoculation with SCC7 and apCAFs. C, Quantitative bar chart of tumor volume in mice from both groups. D, Quantitative bar chart showing the changes in the number of FAP^+^ cells in tumor tissue from both groups. Statistics are shown in mean ± SD (F) accessed by the unpaired t test. *, *P* < 0.05; **, *P* < 0.01; ***, *P* < 0.001; ns, nonsignificant, respectively.

## Data Availability

The data analyzed in this study were obtained from Gene Expression Omnibus (GEO) at GSE164817, GSE164241 and GSE103322. The Graphical abstract was created with BioRender.com.

## References

[1] Johnson DE, Burtness B, Leemans CR, Lui VWY, Bauman JE, Grandis JR. Head and neck squamous cell carcinoma. Nat Rev Dis Primers. 2020;6:00224–223. 10.1038/s41572-020-00224-3 PMC7944998 33243986

[2] Dioguardi M, Caloro GA, Laino L, Alovisi M, Sovereto D, Crincoli V, et al. Circulating miR-21 as a Potential Biomarker for the Diagnosis of Oral Cancer: A Systematic Review with Meta-Analysis. Cancers. 2020;12:12040936. 10.3390/cancers12040936 PMC7226103 32290144

[3] Kumar M, Nanavati R, Modi T, Dobariya C. Oral cancer: Etiology and risk factors: A review. J Cancer Res Ther. 2016;12:186696. 10.4103/0973-1482.186696 27461593

[4] Sung H, Ferlay J, Siegel RL, Laversanne M, Soerjomataram I, Jemal A, et al. Global Cancer Statistics 2020: GLOBOCAN Estimates of Incidence and Mortality Worldwide for 36 Cancers in 185 Countries. CA: A Cancer Journal for Clinicians 2021;71:209–249. 10.3322/caac.21660 33538338

[5] Liu JC, Bhayani M, Kuchta K, Galloway T, Fundakowski C. Patterns of distant metastasis in head and neck cancer at presentation: Implications for initial evaluation. Oral Oncol. 2019;88:131–6. 30616783 10.1016/j.oraloncology.2018.11.023

[6] Elhanani O, Ben-Uri R, Keren L. Spatial profiling technologies illuminate the tumor microenvironment. Cancer Cell. 2023;41:404–20. 36800999 10.1016/j.ccell.2023.01.010

[7] Jin M-Z, Jin W-L. The updated landscape of tumor microenvironment and drug repurposing. Signal Transduct Target Ther. 2020;5:00280–x. 10.1038/s41392-020-00280-x PMC7447642 32843638

[8] Zhi Y, Wang Q, Zi M, Zhang S, Ge J, Liu K, et al. Spatial Transcriptomic and Metabolomic Landscapes of Oral Submucous Fibrosis-Derived Oral Squamous Cell Carcinoma and its Tumor Microenvironment. Advanced Science. 2024;11:06515. 10.1002/advs.202306515 PMC10966560 38229179

[9] Hu C, Zhang Y, Wu C, Huang Q. Heterogeneity of cancer-associated fibroblasts in head and neck squamous cell carcinoma: opportunities and challenges. Cell Death Discovery. 2023;9:01428–01428. 10.1038/s41420-023-01428-8 PMC10102018 37055382

[10] Mazzeo L, Ghosh S, Di Cicco E, Isma J, Tavernari D, Samarkina A, et al. ANKRD1 is a mesenchymal-specific driver of cancer-associated fibroblast activation bridging androgen receptor loss to AP-1 activation. Nature Communications 2024;15:45308-w. 10.1038/s41467-024-45308-w PMC10838290 38310103

[11] McAndrews KM, Chen Y, Darpolor JK, Zheng X, Yang S, Carstens JL, et al. Identification of Functional Heterogeneity of Carcinoma-Associated Fibroblasts with Distinct IL6-Mediated Therapy Resistance in Pancreatic Cancer. Cancer Discov. 2022;12:1580–97. 35348629 10.1158/2159-8290.CD-20-1484 PMC9399904

[12] Affo S, Nair A, Brundu F, Ravichandra A, Bhattacharjee S, Matsuda M, et al. Promotion of cholangiocarcinoma growth by diverse cancer-associated fibroblast subpopulations. Cancer Cell. 2021;39:866-882.e811. 33930309 10.1016/j.ccell.2021.03.012 PMC8241235

[13] Hutton C, Heider F, Blanco-Gomez A, Banyard A, Kononov A, Zhang X, et al. Single-cell analysis defines a pancreatic fibroblast lineage that supports anti-tumor immunity. Cancer Cell. 2021;39:1227-1244.e1220. 34297917 10.1016/j.ccell.2021.06.017 PMC8443274

[14] Li CW, Guo HY, Zhai PS, Yan M, Liu C, Wang XN, et al. Spatial and Single-Cell Transcriptomics Reveal a Cancer-Associated Fibroblast Subset in HNSCC That Restricts Infiltration and Antitumor Activity of CD8<SUP>+</SUP> T Cells. Cancer Res. 2024;84:258–75. 37930937 10.1158/0008-5472.CAN-23-1448 PMC10790129

[15] Bochet L, Lehuédé C, Dauvillier S, Wang YY, Dirat B, Laurent V, et al. Adipocyte-Derived Fibroblasts Promote Tumor Progression and Contribute to the Desmoplastic Reaction in Breast Cancer. Cancer Res. 2013;73:5657–68. 23903958 10.1158/0008-5472.CAN-13-0530

[16] Kojima Y, Acar A, Eaton EN, Mellody KT, Scheel C, Ben-Porath I, et al. Autocrine TGF-β and stromal cell-derived factor-1 (SDF-1) signaling drives the evolution of tumor-promoting mammary stromal myofibroblasts. Proc Natl Acad Sci. 2010;107:20009–14. 21041659 10.1073/pnas.1013805107 PMC2993333

[17] Bu L, Baba H, Yoshida N, Miyake K, Yasuda T, Uchihara T, et al. Biological heterogeneity and versatility of cancer-associated fibroblasts in the tumor microenvironment. Oncogene. 2019;38:4887–901. 30816343 10.1038/s41388-019-0765-y

[18] Hosaka K, Yang Y, Seki T, Fischer C, Dubey O, Fredlund E, et al. Pericyte–fibroblast transition promotes tumor growth and metastasis. Proc Natl Acad Sci. 2016;113:1608384113. 10.1073/pnas.1608384113 PMC5035870 27608497

[19] Tsoumakidou M. The advent of immune stimulating CAFs in cancer. Nat Rev Cancer. 2023;23:258–69. 36807417 10.1038/s41568-023-00549-7

[20] Kürten CHL, Kulkarni A, Cillo AR, Santos PM, Roble AK, Onkar S, et al. Investigating immune and non-immune cell interactions in head and neck tumors by single-cell RNA sequencing. Nat Commun. 2021;12:27619–27614. 10.1038/s41467-021-27619-4 PMC8683505 34921143

[21] van Vlerken-Ysla L, Tyurina YY, Kagan VE, Gabrilovich DI. Functional states of myeloid cells in cancer. Cancer Cell. 2023;41:490–504. 36868224 10.1016/j.ccell.2023.02.009 PMC10023509

[22] Kundu M, Butti R, Panda VK, Malhotra D, Das S, Mitra T, et al. Modulation of the tumor microenvironment and mechanism of immunotherapy-based drug resistance in breast cancer. Mol Cancer. 2024;23:01990–4. 10.1186/s12943-024-01990-4 PMC11075356 38715072

[23] Lin Y, Cai Q, Chen Y, Shi T, Liu W, Mao L, et al. CAFs shape myeloid-derived suppressor cells to promote stemness of intrahepatic cholangiocarcinoma through 5-lipoxygenase. Hepatology. 2022;75:28–42. 34387870 10.1002/hep.32099

[24] Ouyang L, Chang W, Fang B, Qin J, Qu X, Cheng F. Estrogen-induced SDF-1α production promotes the progression of ER-negative breast cancer via the accumulation of MDSCs in the tumor microenvironment. Sci Rep. 2016;6:39541. 27996037 10.1038/srep39541 PMC5172230

[25] Hashimoto O, Yoshida M, Koma Yi, Yanai T, Hasegawa D, Kosaka Y, et al. Collaboration of cancer‐associated fibroblasts and tumour‐associated macrophages for neuroblastoma development. The Journal of Pathology 2016;240:211–223. 10.1002/path.4769 PMC5095779 27425378

[26] Zhao X, Ding L, Lu Z, Huang X, Jing Y, Yang Y, et al. Diminished CD68+ Cancer-Associated Fibroblast Subset Induces Regulatory T-Cell (Treg) Infiltration and Predicts Poor Prognosis of Oral Squamous Cell Carcinoma Patients. Am J Pathol. 2020;190:886–99. 32035062 10.1016/j.ajpath.2019.12.007

[27] Muliaditan T, Caron J, Okesola M, Opzoomer JW, Kosti P, Georgouli M, et al. Macrophages are exploited from an innate wound healing response to facilitate cancer metastasis. Nat Commun. 2018;9:05346–7. 10.1038/s41467-018-05346-7 PMC6063977 30054470

[28] Cassetta L, Fragkogianni S, Sims AH, Swierczak A, Forrester LM, Zhang H, et al. Human Tumor-Associated Macrophage and Monocyte Transcriptional Landscapes Reveal Cancer-Specific Reprogramming, Biomarkers, and Therapeutic Targets. Cancer Cell. 2019;35:588-602.e510. 30930117 10.1016/j.ccell.2019.02.009 PMC6472943

[29] Ubil E, Caskey L, Holtzhausen A, Hunter D, Story C, Earp HS. Tumor-secreted Pros1 inhibits macrophage M1 polarization to reduce antitumor immune response. J Clin Investig. 2018;128:2356–69. 29708510 10.1172/JCI97354 PMC5983338

[30] Elyada E, Bolisetty M, Laise P, Flynn WF, Courtois ET, Burkhart RA, et al. Cross-Species Single-Cell Analysis of Pancreatic Ductal Adenocarcinoma Reveals Antigen-Presenting Cancer-Associated Fibroblasts. Cancer Discov. 2019;9:1102–23. 31197017 10.1158/2159-8290.CD-19-0094 PMC6727976

[31] Sun L, Kang X, Wang C, Wang R, Yang G, Jiang W, et al. Single-cell and spatial dissection of precancerous lesions underlying the initiation process of oral squamous cell carcinoma. Cell Discovery. 2023;9:00532–4. 10.1038/s41421-023-00532-4 PMC10011538 36914617

[32] Puram SV, Tirosh I, Parikh AS, Patel AP, Yizhak K, Gillespie S, et al. Single-Cell Transcriptomic Analysis of Primary and Metastatic Tumor Ecosystems in Head and Neck Cancer. Cell. 2017;171:1611-1624.e1624. 29198524 10.1016/j.cell.2017.10.044 PMC5878932

[33] Wang C, Li Y, Jia L, Kim Jk, Li J, Deng P, et al. CD276 expression enables squamous cell carcinoma stem cells to evade immune surveillance. Cell Stem Cell 2021;28:1597–1613.e1597. 10.1016/j.stem.2021.04.011 PMC8419062 33945793

[34] Williams DW, Greenwell-Wild T, Brenchley L, Dutzan N, Overmiller A, Sawaya AP, et al. Human oral mucosa cell atlas reveals a stromal-neutrophil axis regulating tissue immunity. Cell. 2021;184:4090-4104.e4015. 34129837 10.1016/j.cell.2021.05.013 PMC8359928

[35] Barman PK, Shin JE, Lewis SA, Kang S, Wu D, Wang Y, et al. Production of MHCII-expressing classical monocytes increases during aging in mice and humans. Aging Cell. 2022;21:13701. 10.1111/acel.13701 PMC9577948 36040389

[36] Raudenska M, Balvan J, Hanelova K, Bugajova M, Masarik M. Cancer-associated fibroblasts: Mediators of head and neck tumor microenvironment remodeling. Biochimica et Biophysica Acta (BBA) - Rev Cancer. 2023;1878–89. 10.1016/j.bbcan.2023.188940 37331641

[37] Kumar D, New J, Vishwakarma V, Joshi R, Enders J, Lin F, et al. Cancer-Associated Fibroblasts Drive Glycolysis in a Targetable Signaling Loop Implicated in Head and Neck Squamous Cell Carcinoma Progression. Cancer Res. 2018;78:3769–82. 29769197 10.1158/0008-5472.CAN-17-1076 PMC6050074

[38] Caligiuri G, Tuveson DA. Activated fibroblasts in cancer: Perspectives and challenges. Cancer Cell. 2023;41:434–49. 36917949 10.1016/j.ccell.2023.02.015 PMC11022589

[39] Chhabra Y, Weeraratna AT. Fibroblasts in cancer: Unity in heterogeneity. Cell. 2023;186:1580–609. 37059066 10.1016/j.cell.2023.03.016 PMC11422789

[40] Huang H, Wang Z, Zhang Y, Pradhan RN, Ganguly D, Chandra R, et al. Mesothelial cell-derived antigen-presenting cancer-associated fibroblasts induce expansion of regulatory T cells in pancreatic cancer. Cancer Cell. 2022;40:656-673.e657. 35523176 10.1016/j.ccell.2022.04.011 PMC9197998

[41] Kerdidani D, Aerakis E, Verrou K-M, Angelidis I, Douka K, Maniou M-A, et al. Lung tumor MHCII immunity depends on in situ antigen presentation by fibroblasts. J Exp Med. 2022;219:0815. 10.1084/jem.20210815 PMC8764966 35029648

[42] Costa A, Kieffer Y, Scholer-Dahirel A, Pelon F, Bourachot B, Cardon M, et al. Fibroblast Heterogeneity and Immunosuppressive Environment in Human Breast Cancer. Cancer Cell. 2018;33:463-479.e410. 29455927 10.1016/j.ccell.2018.01.011

[43] Biffi G, Tuveson DA. Diversity and Biology of Cancer-Associated Fibroblasts. Physiol Rev. 2021;101:147–76. 32466724 10.1152/physrev.00048.2019 PMC7864232

[44] Öhlund D, Handly-Santana A, Biffi G, Elyada E, Almeida AS, Ponz-Sarvise M, et al. Distinct populations of inflammatory fibroblasts and myofibroblasts in pancreatic cancer. J Exp Med. 2017;214:579–96. 28232471 10.1084/jem.20162024 PMC5339682

[45] Tang PCT, Chung JYF, Xue VWw, Xiao J, Meng XM, Huang XR, et al. Smad3 Promotes Cancer‐Associated Fibroblasts Generation via Macrophage–Myofibroblast Transition. Advanced Science 2021;9:01235. 10.1002/advs.202101235 PMC8728853 34791825

[46] Huang X, He C, Hua X, Kan A, Mao Y, Sun S, et al. Oxidative stress induces monocyte-to-myofibroblast transdifferentiation through p38 in pancreatic ductal adenocarcinoma. Clin Transl Med. 2020;10:41. 10.1002/ctm2.41 PMC7403727 32508052

[47] Pazolli E, Alspach E, Milczarek A, Prior J, Piwnica-Worms D, Stewart SA. Chromatin Remodeling Underlies the Senescence-Associated Secretory Phenotype of Tumor Stromal Fibroblasts That Supports Cancer Progression. Cancer Res. 2012;72:2251–61. 22422937 10.1158/0008-5472.CAN-11-3386 PMC3605047

[48] Martinez-Outschoorn UE, Balliet RM, Rivadeneira D, Chiavarina B, Pavlides S, Wang C, et al. Oxidative stress in cancer associated fibroblasts drives tumor-stroma co-evolution. Cell Cycle. 2014;9:3276–96. 10.4161/cc.9.16.12553 PMC3041164 20814239

[49] Quante M, Tu SP, Tomita H, Gonda T, Wang SSW, Takashi S, et al. Bone Marrow-Derived Myofibroblasts Contribute to the Mesenchymal Stem Cell Niche and Promote Tumor Growth. Cancer Cell. 2011;19:257–72. 21316604 10.1016/j.ccr.2011.01.020 PMC3060401

[50] Mitra AK, Zillhardt M, Hua Y, Tiwari P, Murmann AE, Peter ME, et al. MicroRNAs Reprogram Normal Fibroblasts into Cancer-Associated Fibroblasts in Ovarian Cancer. Cancer Discov. 2012;2:1100–8. 23171795 10.1158/2159-8290.CD-12-0206 PMC3685866

[51] Fang T, Lv H, Lv G, Li T, Wang C, Han Q, et al. Tumor-derived exosomal miR-1247-3p induces cancer-associated fibroblast activation to foster lung metastasis of liver cancer. Nat Commun. 2018;9:02583–02580. 10.1038/s41467-017-02583-0 PMC5768693 29335551

[52] Zhang D, Wang Y, Shi Z, Liu J, Sun P, Hou X, et al. Metabolic Reprogramming of Cancer-Associated Fibroblasts by IDH3α Downregulation. Cell Rep. 2015;10:1335–48. 25732824 10.1016/j.celrep.2015.02.006

[53] Kang I, Bucala R. The immunobiology of MIF: function, genetics and prospects for precision medicine. Nat Rev Rheumatol. 2019;15:427–37. 31197253 10.1038/s41584-019-0238-2

[54] Sumaiya K, Langford D, Natarajaseenivasan K, Shanmughapriya S. Macrophage migration inhibitory factor (MIF): A multifaceted cytokine regulated by genetic and physiological strategies. Pharmacol Ther. 2022;233–52. 10.1016/j.pharmthera.2021.108024 34673115

[55] Tessaro FHG, Ko EY, De Simone M, Piras R, Broz MT, Goodridge HS, et al. Single-cell RNA-seq of a soft-tissue sarcoma model reveals the critical role of tumor-expressed MIF in shaping macrophage heterogeneity. Cell Rep. 2022;39:110977. 35732118 10.1016/j.celrep.2022.110977 PMC9249098

[56] Biffi G, Oni TE, Spielman B, Hao Y, Elyada E, Park Y, et al. IL1-Induced JAK/STAT Signaling Is Antagonized by TGFβ to Shape CAF Heterogeneity in Pancreatic Ductal Adenocarcinoma. Cancer Discov. 2019;9:282–301. 30366930 10.1158/2159-8290.CD-18-0710 PMC6368881

[57] Somerville TDD, Biffi G, Daßler-Plenker J, Hur SK, He X-Y, Vance KE, et al. Squamous trans-differentiation of pancreatic cancer cells promotes stromal inflammation. eLife. 2020;9:53381. 10.7554/eLife.53381 PMC7200154 32329713

[58] Sebastian A, Hum NR, Martin KA, Gilmore SF, Peran I, Byers SW, et al. Single-Cell Transcriptomic Analysis of Tumor-Derived Fibroblasts and Normal Tissue-Resident Fibroblasts Reveals Fibroblast Heterogeneity in Breast Cancer. Cancers. 2020;12:1307. 32455670 10.3390/cancers12051307 PMC7281266

[59] Kieffer Y, Hocine HR, Gentric G, Pelon F, Bernard C, Bourachot B, et al. Single-Cell Analysis Reveals Fibroblast Clusters Linked to Immunotherapy Resistance in Cancer. Cancer Discov. 2020;10:1330–51. 32434947 10.1158/2159-8290.CD-19-1384

[60] Lee IK, Noguera-Ortega E, Xiao Z, Todd L, Scholler J, Song D, et al. Monitoring Therapeutic Response to Anti-FAP CAR T Cells Using [18F]AlF-FAPI-74. Clin Cancer Res. 2022;28:5330–42. 35972732 10.1158/1078-0432.CCR-22-1379 PMC9771904

[61] Wang J, Tannous BA, Poznansky MC, Chen H. CXCR4 antagonist AMD3100 (plerixafor): From an impurity to a therapeutic agent. Pharmacol Res. 2020;159:105010. 32544428 10.1016/j.phrs.2020.105010

[62] Jia X, Xi J, Tian B, Zhang Y, Wang Z, Wang F, et al. The Tautomerase Activity of Tumor Exosomal MIF Promotes Pancreatic Cancer Progression by Modulating MDSC Differentiation. Cancer Immunol Res. 2024;12:72–90. 37956411 10.1158/2326-6066.CIR-23-0205

[63] Yan L, Wu M, Wang T, Yuan H, Zhang X, Zhang H, et al. Breast Cancer Stem Cells Secrete MIF to Mediate Tumor Metabolic Reprogramming That Drives Immune Evasion. Cancer Res. 2024;84:1270–85. 38335272 10.1158/0008-5472.CAN-23-2390

[64] Liu L, Wang J, Wang Y, Chen L, Peng L, Bin Y, et al. Blocking the MIF-CD74 axis augments radiotherapy efficacy for brain metastasis in NSCLC via synergistically promoting microglia M1 polarization. J Exp Clin Cancer Res. 2024;43:128. 38685050 10.1186/s13046-024-03024-9 PMC11059744

