# Supplementary material for: Myeloid cell-derived apCAFs promote HNSCC progression by regulating proportion of CD4+ and CD8+ T cells
Source: J Exp Clin Cancer Res. 2025 Jan 31;44:33. doi: 10.1186/s13046-025-03290-1 (PMC11783918; doi:10.1186/s13046-025-03290-1)
Supplement: Supplementary file 1 — Supplementary Material 1: Fig. 1. HE staining of tumor tissues from different HNSCC models in both patients and mice. A, Hematoxylin and eosin (H&E) stained histological sections of tumor tissues from patients with OSCC used in this study. B, H&E staining histological sections of OSCC samples induced by 4NQO in mice. C, H&E staining histological sections of murine transplant tumors following inoculation with the mouse OSCC cell line SCC7. Fig. 2. Successfully established a myeloid cell-specific fluorescent reporter mouse and an OSCC model. A, Tissue specimens of normal mouse tongue and 4NQO-induced OSCC in mouse tongue. B, Genotyping results for Lyz2-Cre/ROSA-mTmG mice. C, Fluorescence identification results of spleen in Lyz2-Cre;ROSA-mTmG mice. Fig. 3. Single-cell data and TCGA database analysis results. A, the expression levels of different fibroblast-associated markers (COL3A1, COL1A1, PDGFRB, FAP) and MHC class II-related markers (HLA-DPB1, HLA-DRA, HLA-DPA1, CD74) across three CAFs subpopulations in HNSCC. B, Pseudotime analysis results of epithelial cells and CAFs in human and mouse HNSCC. C, ACTA2 expression results from bulk RNA sequencing data of HNSCC from the TCGA database. Data are presented as range and mean ± SEM, analyzed by the Wilcoxon test. D, Survival analysis of patients with HNSCC shows that high ACTA2 expression is not associated with overall survival rates. Fig. 4. The macrophage clearance leads to an increase in the CD8+ T/CD4+ T ratio. A, Expression changes of macrophage markers and apCAF-related markers in THP1 cells after stimulation with different concentrations of MCSF for 6 h. B and E, Flow cytometry dot plots and quantitative results showing the changes in the number of FAP+ cells after macrophage depletion. C and F, Flow cytometry dot plots and quantitative results showing the changes in the number of CD4+ T cells following macrophage depletion. D and G, Flow cytometry dot plots and quantitative results showing the changes in the numb [file 13046_2025_3290_MOESM1_ESM.pdf]

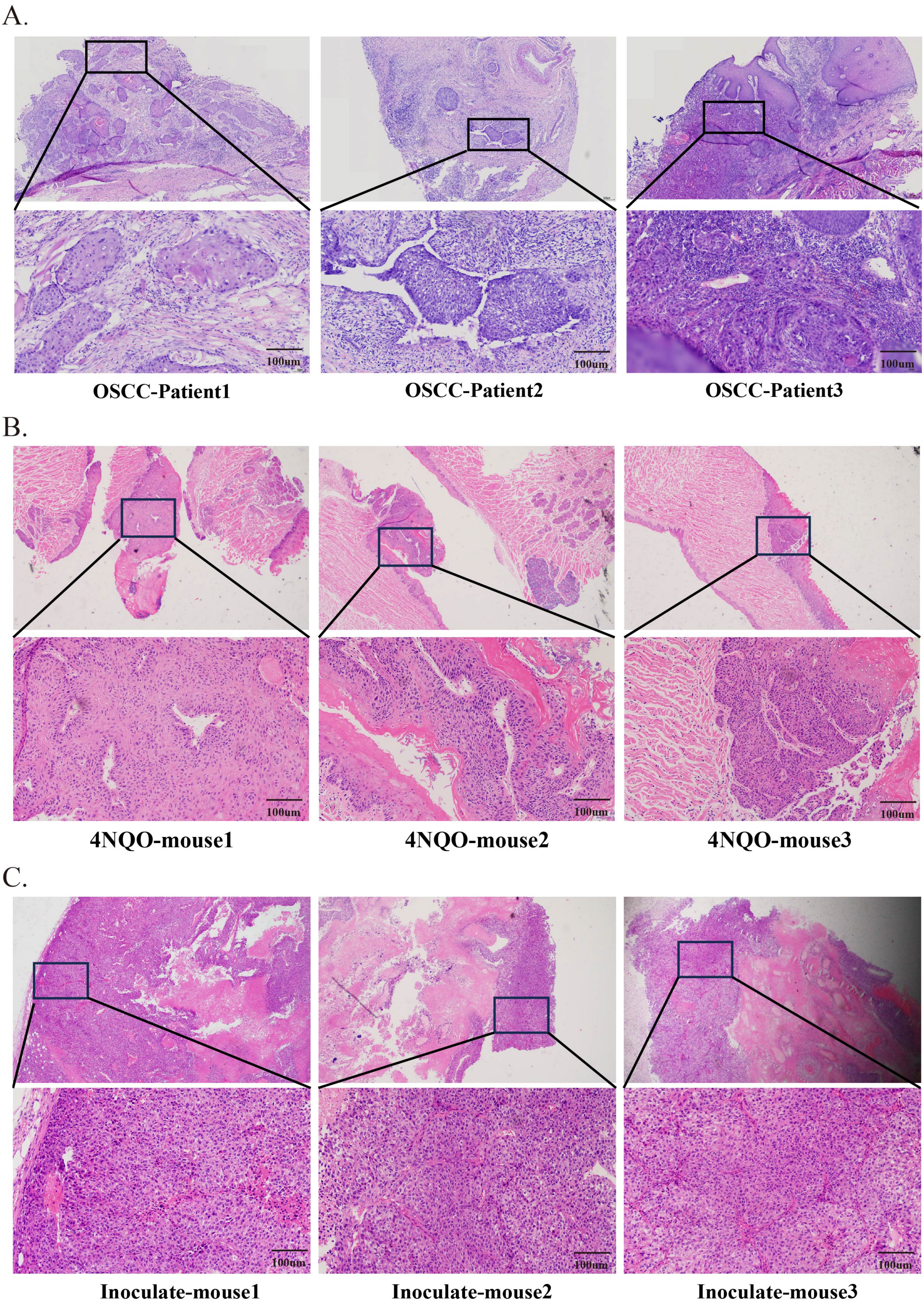
A.
100um
100um
100um
OSCC-Patient1
OSCC-Patient2
OSCC-Patient3
B.
100um
100um
100um
4NQO-mouse1
4NQO-mouse2
4NQO-mouse3
C.
100um
100um
100um
Inoculate-mouse1
Inoculate-mouse2
Inoculate-mouse3

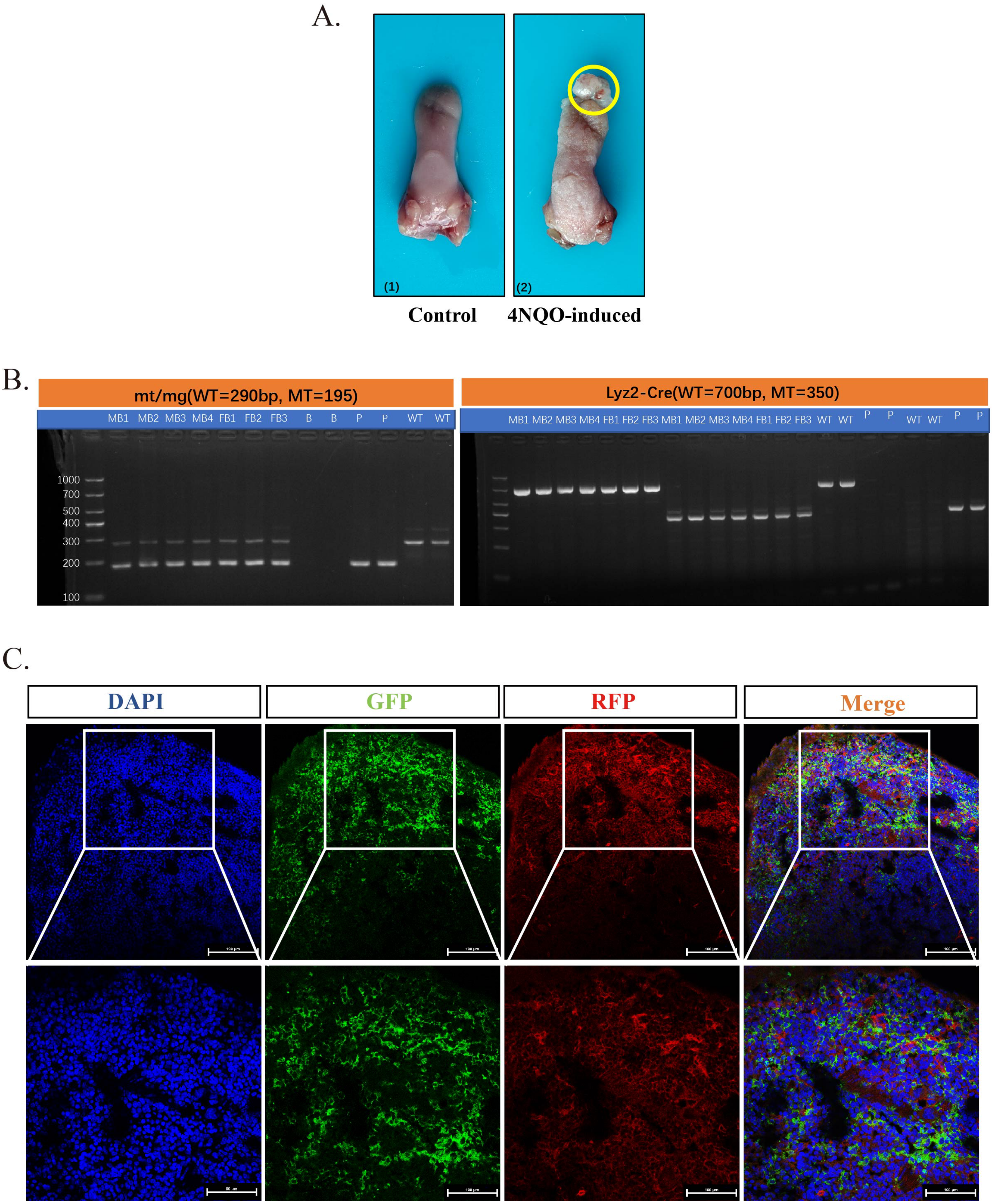
A.
(1)
(2)
Control
4NQO-induced
B.
mt/mg(WT=290bp, MT=195)
MB1 MB2 MB3 MB4 FB1 FB2 FB3 B B P P WT WT
1000
700
500
400
300
200
100
Lyz2-Cre(WT=700bp, MT=350)
MB1 MB2 MB3 MB4 FB1 FB2 FB3 MB1 MB2 MB3 MB4 FB1 FB2 FB3 WT WT P P WT WT P P
C.
DAPI
GFP
RFP
Merge

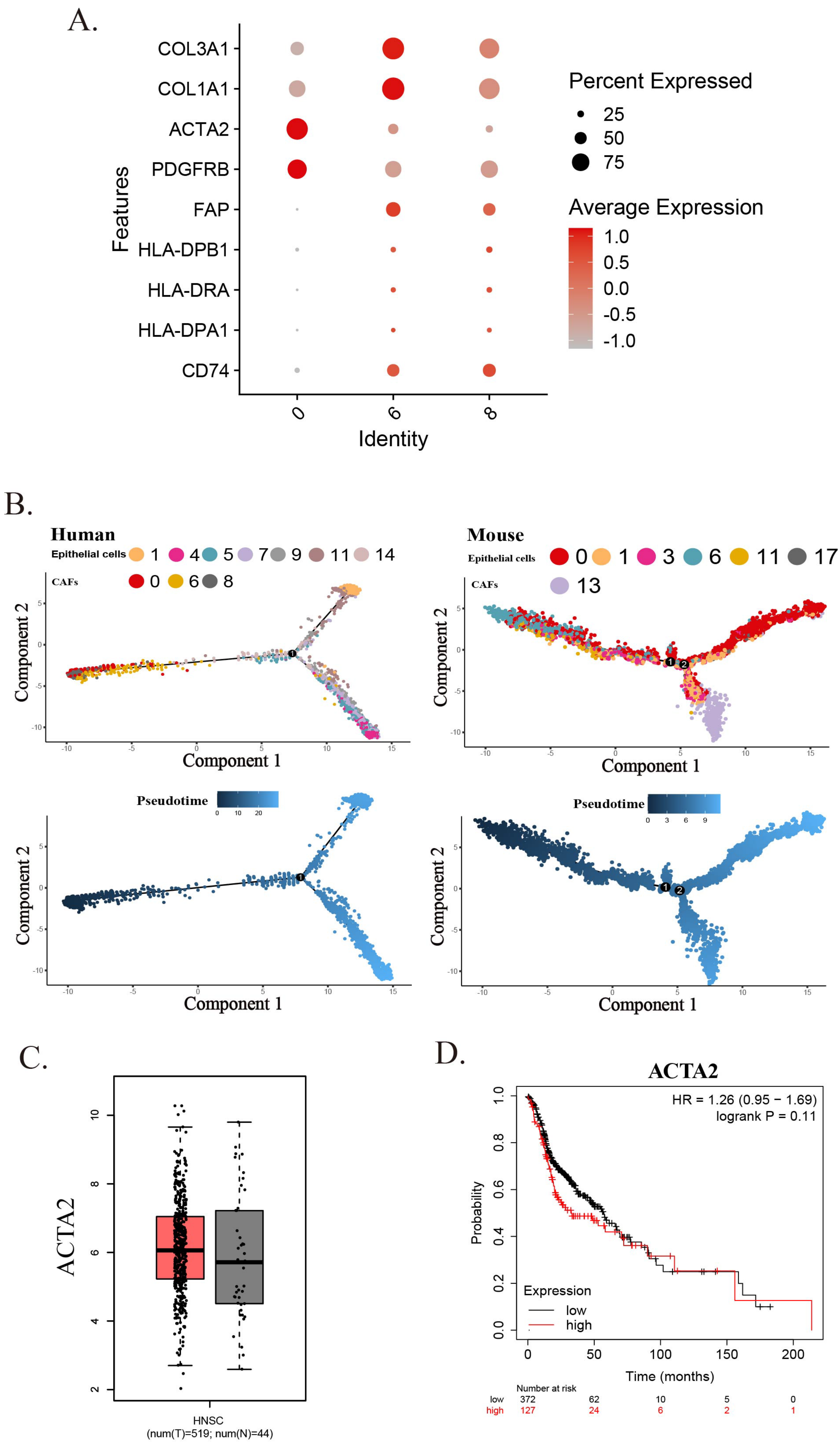
A.
COL3A1
COL1A1
ACTA2
PDGFRB
FAP
HLA-DPB1
HLA-DRA
HLA-DPA1
CD74
Features
0
6
8
Identity
Percent Expressed
25
50
75
Average Expression
1.0
0.5
0.0
-0.5
-1.0
B.
Human
Epithelial cells 1 4 5 7 9 11 14
CAFs 0 6 8
Component 2
Component 1
Pseudotime
0 10 20
Mouse
Epithelial cells 0 1 3 6 11 17
CAFs 13
Pseudotime
0 3 6 9
C.
ACTA2
HNSC
(num(T)=519; num(N)=44)
D.
ACTA2
HR = 1.26 (0.95 − 1.69)
logrank P = 0.11
Probability
Expression
low
high
Time (months)
Number at risk
low 372 62 10 5 0
high 127 24 6 2 1

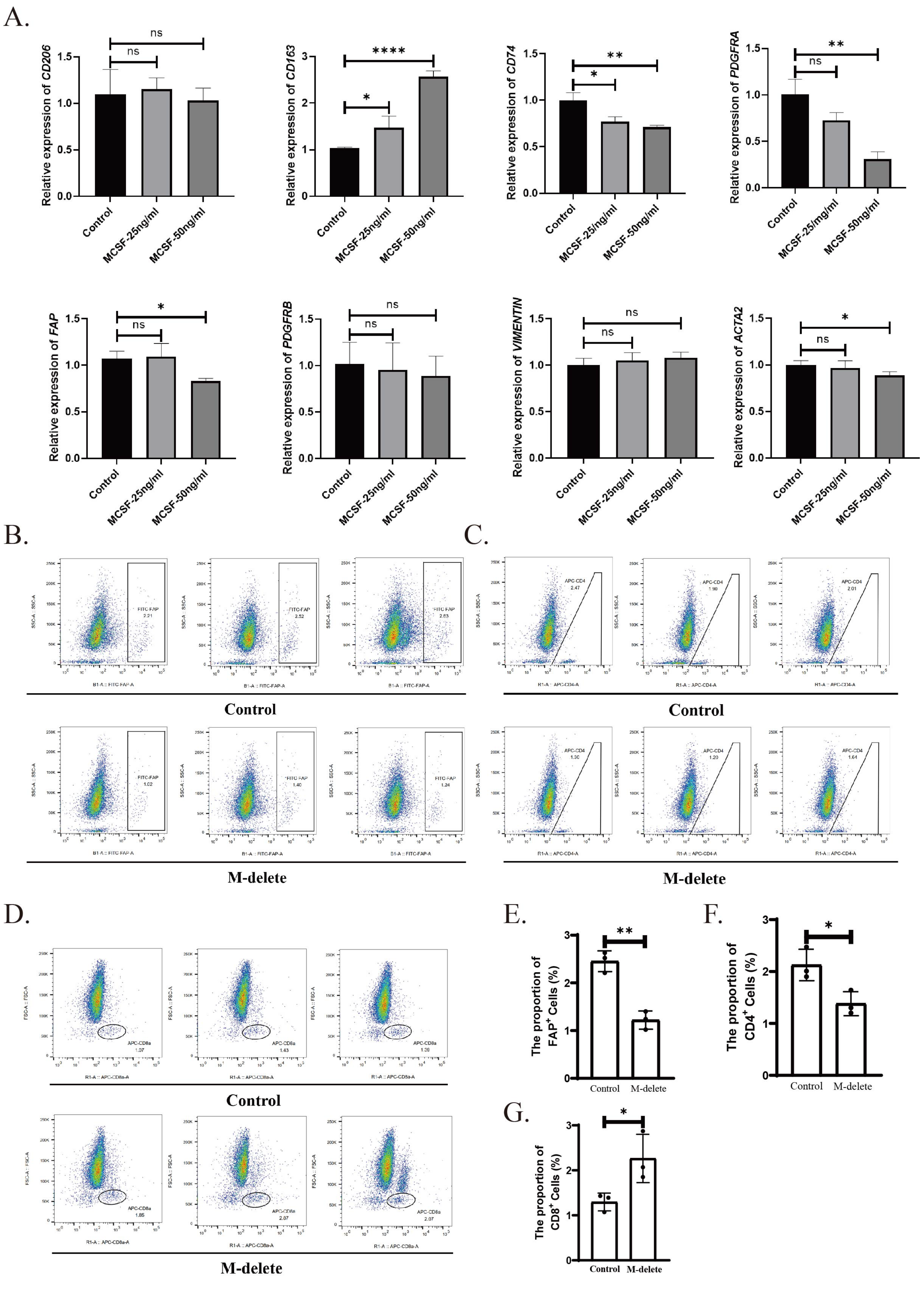

A.
Relative expression of CD206
Relative expression of CD163
Relative expression of CD74
Relative expression of PDGFRA
Relative expression of FAP
Relative expression of PDGFRB
Relative expression of VIMENTIN
Relative expression of ACTA2
Control
MCSF-25ng/ml
MCSF-50ng/ml
MCSF-25/ng/ml
MCSF-25/mg/ml
ns
*
**
****
B.
C.
Control
M-delete
D.
E.
F.
G.
The proportion of FAP+ Cells (%)
The proportion of CD4+ Cells (%)
The proportion of CD8+ Cells (%)

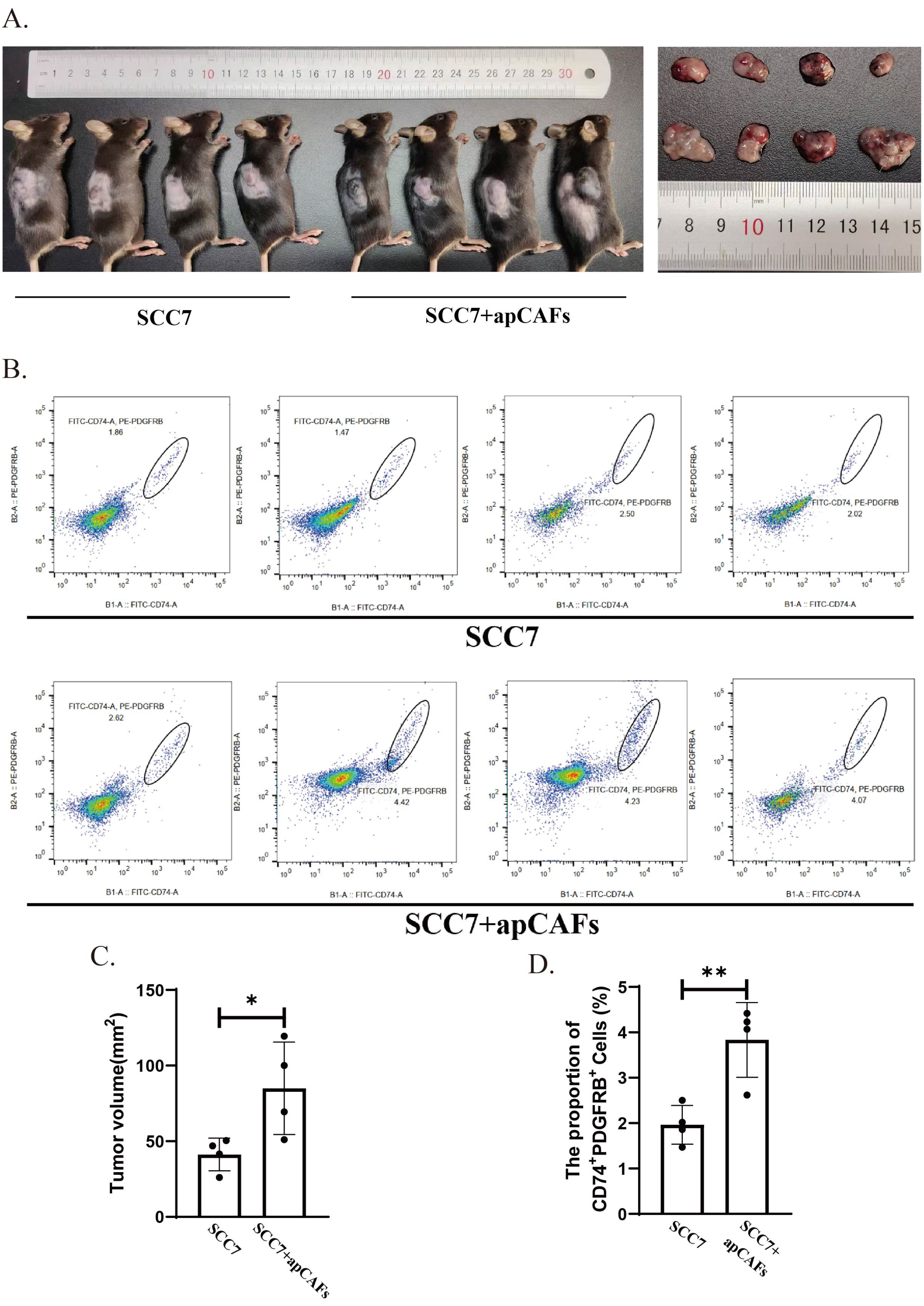
A.
SCC7
SCC7+apCAFs
B.
FITC-CD74-A, PE-PDGFRB
1.86
FITC-CD74-A, PE-PDGFRB
1.47
FITC-CD74, PE-PDGFRB
2.50
FITC-CD74, PE-PDGFRB
2.02
B2-A :: PE-PDGFRB-A
B1-A :: FITC-CD74-A
SCC7
FITC-CD74-A, PE-PDGFRB
2.62
FITC-CD74, PE-PDGFRB
4.42
FITC-CD74, PE-PDGFRB
4.23
FITC-CD74, PE-PDGFRB
4.07
B2-A :: PE-PDGFRB-A
B1-A :: FITC-CD74-A
SCC7+apCAFs
C.
Tumor volume(mm2)
*
SCC7
SCC7+apCAFs
D.
The proportion of CD74+PDGFRB+ Cells (%)
**
SCC7
SCC7+ apCAFs
